# Single-nuclei analysis reveals depot-specific transcriptional heterogeneity and depot-specific cell types in adipose tissue of dairy cows

**DOI:** 10.3389/fcell.2022.1025240

**Published:** 2022-10-14

**Authors:** Tainara C. Michelotti, Brent R. Kisby, Lauryn S. Flores, Alexandra P. Tegeler, Mohamed Fokar, Chiquito Crasto, Bruno C. Menarim, Shavahn C. Loux, Clarissa Strieder-Barboza

**Affiliations:** ^1^ Department of Veterinary Sciences, Davis College of Agricultural Sciences and Natural Resources, Texas Tech University, Lubbock, TX, United States; ^2^ Department of Pharmacology and Neuroscience, Texas Tech University Health Science Center, Lubbock, TX, United States; ^3^ Center for Biotechnology and Genomics, Texas Tech University, Lubbock, TX, United States; ^4^ Department of Computer Science, Whitacre College of Engineering, Texas Tech University, Lubbock, TX, United States; ^5^ Department of University Studies, Texas Tech University, Lubbock, TX, United States; ^6^ Gluck Equine Research Center, Department of Veterinary Science, University of Kentucky, Lexington, KY, United States; ^7^ School of Veterinary Medicine, Texas Tech University, Amarillo, TX, United States

**Keywords:** single-nuclei analysis, dairy cow, adipose tissue metabolism, depot differences, progenitor cell

## Abstract

Adipose tissue (AT) is an endocrine organ with a central role on whole-body energy metabolism and development of metabolic diseases. Single-cell and single-nuclei RNA sequencing (scRNA-seq and snRNA-seq, respectively) analyses in mice and human AT have revealed vast cell heterogeneity and functionally distinct subtypes that are potential therapeutic targets to metabolic disease. In periparturient dairy cows, AT goes through intensive remodeling and its dysfunction is associated with metabolic disease pathogenesis and decreased productive performance. The contributions of depot-specific cells and subtypes to the development of diseases in dairy cows remain to be studied. Our objective was to elucidate differences in cellular diversity of visceral (VAT) and subcutaneous (SAT) AT in dairy cows at the single-nuclei level. We collected matched SAT and VAT samples from three dairy cows and performed snRNA-seq analysis. We identified distinct cell types including four major mature adipocytes (AD) and three stem and progenitor cells (ASPC) subtypes, along with endothelial cells (EC), mesothelial cells (ME), immune cells, and pericytes and smooth muscle cells. All major cell types were present in both SAT and VAT, although a strong VAT-specificity was observed for ME, which were basically absent in SAT. One ASPC subtype was defined as adipogenic (*PPARG*+) while the other two had a fibro-adipogenic profile (*PDGFRA+*). We identified vascular and lymphatic EC subtypes, and different immune cell types and subtypes in both SAT and VAT, i.e., macrophages, monocytes, T cells, and natural killer cells. Not only did VAT show a greater proportion of immune cells, but these visceral immune cells had greater activation of pathways related to immune and inflammatory response, and complement cascade in comparison with SAT. There was a substantial contrast between depots for gene expression of complement cascade, which were greatly expressed by VAT cell subtypes compared to SAT, indicating a pro-inflammatory profile in VAT. Unprecedently, our study demonstrated cell-type and depot-specific heterogeneity in VAT and SAT of dairy cows. A better understanding of depot-specific molecular and cellular features of SAT and VAT will aid in the development of AT-targeted strategies to prevent and treat metabolic disease in dairy cows, especially during the periparturient period.

## Graphical Abstract

Graphical summary. Transcriptional profile (differently expressed genes - DEG) of distinct cell types and subtypes obtained through single-nuclei RNA sequencing analysis of matched omental visceral (VAT) and abdominal subcutaneous adipose tissues (SAT) from dairy cows (n = 3). Genes following each cell types (*e.g.* adipocytes, macrophages) represent signature genes through they were identified. Column on the right includes the depot in which each cell type is more abundant. ‘+’ and ‘−’ represent the expression and lack of expression, respectively, for that specific gene.
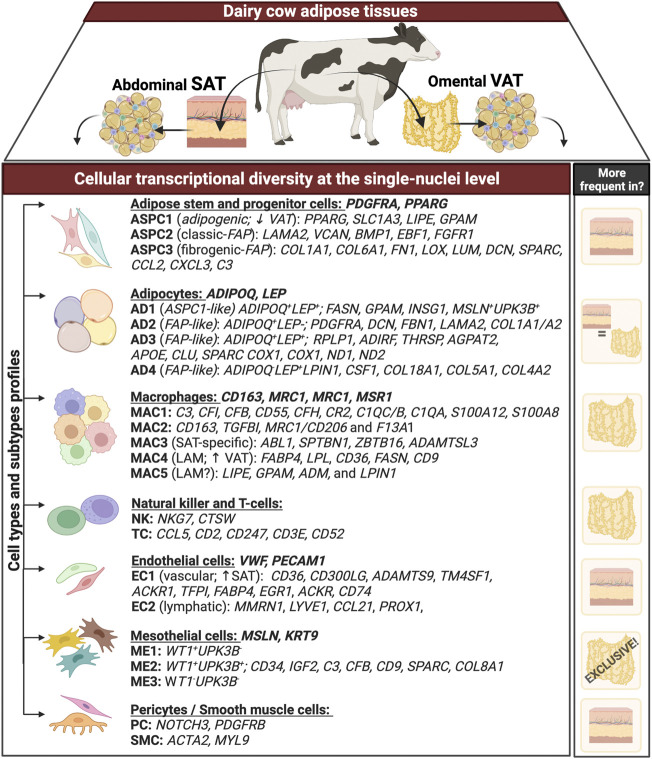

## Introduction

Adipose tissue (AT) is a central metabolic organ that regulates whole-body energy homeostasis. In dairy cows, abnormal AT responses to changes in the endocrine status and energy balance are associated with the development of metabolic disease (Contreras and Sordillo, 2011; De Koster and Opsomer, 2013; Contreras et al., 2017; Contreras et al., 2018). Features of AT dysfunction in dairy cows include dysregulated inflammation with increased infiltration of macrophages, excessive adipocyte lipolysis, and persistent insulin resistance (De Koster et al., 2017; Contreras et al., 2018). Decreased adipogenic capacity and changes in AT extracellular matrix (ECM) function and deposition have also been implicated as important features of AT dysfunction in humans and mice with metabolic disease (Muir et al., 2016; Baker et al., 2017; Strieder-Barboza et al., 2020), but scarcely reported in dairy cows.

Adipose tissue surrounding abdominal viscera in the mesentery and omentum, also known as visceral AT (VAT), is structurally and functionally different from that present in subcutaneous areas (subcutaneous adipose tissue - SAT). In dairy cows, VAT exhibits decreased adipocyte size and adipogenic capacity (lower number of adipocyte progenitor cells) and has an increased pro-inflammatory reaction in response to metabolic disease compared with SAT, which has larger adipocytes and more robust lipolytic responses (Contreras et al., 2015; Depreester et al., 2018; Strieder-Barboza et al., 2019). These depot-specific structural and functional responses to metabolic disease suggest differential regulation of systemic metabolic function by the VAT and SAT depots in dairy cows. This may have important implications for understanding the pathogenesis of disease in dairy cows and developing novel cell-targeted interventions to prevent and treat metabolic disease.

Recent studies using single-cell and single-nuclei RNA sequencing analysis (scRNA-seq and snRNA-seq, respectively) of AT in humans and mice revealed significant cell heterogeneity and functionally distinct subpopulations of adipocyte progenitor cells, endothelial cells, mature adipocytes, and among other cell types (Vijay et al., 2020; Emont et al., 2022; Strieder-Barboza et al., 2022). Furthermore, AT presents depot- and disease-specific molecularly and functionally distinct subpopulations, with important implications in the development of metabolic diseases. For example, different subpopulations of stromal cells have been shown to inhibit or enhance inflammation*,* lipolysis, and adipogenesis in the AT (Burl et al., 2018; Hepler et al., 2018; Schwalie et al., 2018; Merrick et al., 2019; Vijay et al., 2020; Sárvári et al., 2021). These findings point to a complex network of cell subpopulations that regulate AT metabolic function in a depot-specific manner.

Elucidating the molecular and cellular features of SAT and VAT that generate differential metabolic effects could help in understanding how specific AT depots contribute to disease development in dairy cows, especially during the periparturient period. Our objective for this study was to elucidate differences in cellular diversity of VAT and SAT in dairy cows at the single-nuclei level. We revealed that VAT and SAT from dairy cows are highly heterogeneous and contain depot-specific cell subtypes. These findings highlight the uniqueness of AT as a target organ for modulating systemic metabolism and preventing metabolic diseases in dairy cows.

## Materials and methods

### Nuclei isolation and single-nuclei RNA sequencing in adipose tissue samples

Visceral adipose tissue was collected from greater omentum and SAT from the right flank of the same three Holstein dairy cows in a local abattoir (Supplementary Figure S1A). Animals were randomly selected from a group of adult Holstein cows brought to the harvest plant. We did not have access to any data about these animals. The samples were collected within 15 min after exsanguination and snap frozen in liquid nitrogen, transported to the laboratory, and stored at −80°C until further processing. Nuclei-isolation from bovine VAT and SAT was adapted from a previous protocol with brain tissue (Krishnaswami et al., 2016). Briefly, 500 mg of each cryopreserved VAT or SAT were washed with sterile RNase free cold 1X PBS (10XPBS buffer, pH 7.4, Invitrogen, Cat. No. AM9625 diluted 1:10 with Nuclease-free water, Invitrogen, Cat. No. AM9932), then minced with a scalpel using a petri dish on ice (Supplementary Figures S1B–C). Samples were homogenized in a precooled 15 ml glass dounce homogenizer (10 strokes with pestle A followed by 15 strokes with pestle B) with 1.5 ml of homogenization buffer composed by 5 mM MgCl_2_ (Invitrogen, Cat. No. AM9530G), 10 mM Tris Buffer (pH 8.0) (Invitrogen, Cat. No. AM9855G), 25 mM KCl (Invitrogen, Cat. No. AM9640G), 250 mM sucrose (Sigma-Aldrich, Cat. No. S0389-500 g), 1X protease inhibitor (Roche, Cat. No. 11836170001), 1 µM DL-Dithiothreitol solution (DTT, Sigma-Aldrich, Cat. No. 646563-10X), 0.4 U/µl Ribolock RNase Inhibitor (40 U/μl) (ThermoScientific, Cat. No. EO0381), 0.2 U/µl Superasin (20 U/µl) (Invitrogen, Cat. No. AM2696), and 0.1% Triton X-100 (Sigma-Aldrich, Cat. No. T8787-100ML). Samples were then strained with pre-wet 100 µm and 40 µm filters into a 50 ml conical tube. Next, each sample was transferred to two 1.5 ml pre-chilled microcentrifuge RNase free tubes and centrifuged at 500 × g, 4°C for 5 min. Supernatant was pipetted off leaving approximately 50 µL containing the nuclei pellet, which was resuspended in 500 µl of 1% BSA-PBS (Bovine serum albumin, Sigma-Aldrich, Cat. No. A7030-10G) including 0.2 U/μl of Ribolock RNase Inhibitors. Before proceeding with fluorescence activated cell sorting (FACS), a subsample of nuclei was used to assess the overall quality of the nuclei by staining with trypan blue and visualized by phase-contrast light microscopy (Supplementary Figure S1C). To enable the sorting of the nuclei, we immune-stained the nuclei samples with propidium iodide (PI) (Invitrogen, Cat. No. V13241, 10 µg/ml) in a 1:100 dilution, leaving approximately 20–30 µl of sample to be used as unstained control. Each sample was transferred into a pre-coated 5 ml polystyrene flow cytometry tube and sorted using a 100 µM nozzle in a BD FACSAria II Cell Sorter (BD Biosciences, Franklin Lakes, NJ, United States). Sorting strategy included doublet discrimination and selection of intact nuclei by sub-gating on PI stanning (Supplementary Figure S1D). PI+ nuclei was sorted directly into a 1.5 ml microcentrifuge tube containing 20 µl of 1% BSA-PBS and 0.2 U/μl of Ribolock RNase Inhibitors. After nuclei-isolation, samples were centrifuged at 100 × g, 4°C for 6 min, and the supernatant was pipetted off leaving approximately 50 µl of resuspended nuclei sample. Single nuclei suspensions were subjected to final nuclei counting on an automated cell counter (Countess 2, Life Technologies Inc., Carlsbad, CA, United States) and diluted to a concentration of 700–1,000 nuclei/µl. 3′ single nuclei libraries were generated following manufacturer’s user guide: 10X Genomics Chromium Next GEM Single Cell 3′ Reagent Kits v3.1 (Dual Index). Final library quality was resolved using DS 5000 HS assay kit using Tape Station 4200 (Agilent Technologies, Santa Clara, CA, United States). The libraries were quantified using Qubit dsDNA HS assay kit on Qubit Fluorometer version 2.0 (Life Technologies Inc., Carlsbad, CA, United States) (Supplementary Figure S1E).

### Single-nuclei RNA sequencing and data analysis

The pooled nuclei libraries were subjected to 150 bp paired-end sequencing according to the manufacturer’s protocol (Illumina NovaSeq 6000) (Supplementary Figure S1F). Bcl2fastq2 Conversion Software (Illumina) was used to generate de-multiplexed Fastq files, and the CellRanger Pipeline (10X Genomics) was used to align reads and generate count matrices. Data analysis was performed using the scRNA-Seq package Seurat v3.1.4 (Stuart et al., 2019) in the R environment (version 4.1.3), following recommended practice for scRNA-Seq analysis, including quality control, normalization, and scaling of data, feature selection, dimensionality reduction, clustering, and visualization of data (Supplementary Figure S1G). Data quality control was performed by: 1) removing genes observed in fewer than 3 cells to avoid random noise; 2) filtering nuclei with a minimum gene count of 300 and a maximum of 6,000 genes; and 3) setting a threshold of <15% for mitochondrial gene expression. In Seurat, data were normalized using the “NormalizeData” function while “CellCycleScoring” was used to classify nuclei regarding cell cycle stage and assign respective scores to each nucleus. Variability was regressed out by the difference between G2M and S phase score based on the gene expression of cell cycle genes by scaling and centering the residuals as implemented in the function “ScaleData”. Using the “FindVariableFeatures” function in Seurat, we identified 8000 highly variable genes. The scaled and normalized expression data of respective genes served as input for a principal component analysis (PCA), and the top 30 dimensions were used to plot the variability between cells in a two-dimensional diagram by means of the Uniform Manifold Approximation and Projection (UMAP) procedure to reduce the dimensionality of the data. Cells were clustered into subpopulations according to the same dimensions using the “FindClusters” function with a 0.6 resolution, which is a graph-based clustering approach. The top genes in each cluster were identified in comparison with all the other clusters using the “FindMarkers” function while keeping a cutoff of Log_2_FoldChange > 0.5 and *Adjusted p value* < 0.05, calculated based on Bonferroni correction using all genes in the dataset. Genes expressed in a minimum of 10% of nuclei in the test population were considered for analysis. Cell types were assigned manually to each cluster based on known expressions of signature genes. Clusters with nuclei that expressed marker genes for a specific cell type at the highest levels were assigned the corresponding cell type label (Supplementary Table S1). The subclustering of major cell subtypes was created from the “Subset” function of Seurat. Dimensional reduction, UMAP, and clustering were performed as described above, except for resolution, which was increased to 0.8. Finally, using the “Subset” and “FindMarkers” function, we compared each cluster between SAT and VAT and identified the top genes following the same specifications as described above. Overall, the cell type-specific contrasts between depots were analyzed for Gene ontology (GO) enrichment pathways. Analyses were performed on the entire gene list using clusterProfiler (version 4.0.0) in the R environment (version 4.1.3). Genes were evaluated for enrichment in GO Biological Process (BP), Molecular Function (MF), and Cellular Component (CC) using *Adjusted p value* < 0.05 after Benjamini–Hochberg correction. All pathways and *Adjusted p values* can be found in Supplementary Table S2. Contrast between different adipose stem and progenitor cells and mature adipocytes populations were also analyzed for GO enrichment pathways following the same specifications described above, and results can be found in Supplementary Tables S3, S4, respectively. Using available datasets, we compared bovine AT snRNA-seq with human AT scRNA-seq. Human AT scRNA-seq was handled as described above, using the same parameters of quality control, normalization, scaling of data, and feature selection. To compare the different datasets, we identified anchors using the “FindIntegrationAnchors” function, then performed the integration analysis using the “IntegrateData” function of Seurat. Dimensionality reduction, clustering, and visualization of data followed the parameters mentioned above. UMAPs were plotted using the group. by = “orig.ident” and split. by = “orig.ident” statements in order to highlight contrast between studies.

### Tissue processing and cell culture

Adipose tissue samples were collected from randomly selected Holstein cows in a local abattoir and stromal vascular fraction (SVF) isolated as previously reported (Strieder-Barboza et al., 2018; Strieder-Barboza and Contreras, 2019). Briefly, abdominal SAT and omental VAT were collected in Krebs-Ringer modified buffer (KRB) at 37°C supplemented with HEPES 10 M (pH = 7.3, ThermoScientific, Cat. No. J16924. K2) and gentamicin (50 mg/ml, Sigma-Aldrich, Cat. No. G1397-10 ml). Approximately 5 g of each sample was digested in 15 ml of collagenase type II solution (2 mg/ml; Gibco, Cat. No. 17101-015) for 1 h at 37°C in a shaker (490 rpm). Tissue digesta was sequentially filtered through 100- and 40-µm cell strainers (FisherScientific, Cat. No. 22-363-549 and 22-363-547) using 5 ml of KRB with 4% BSA (FisherScientific, Cat. No. BP1600-100), and filtrate centrifuged (800 × g, 10 min, room temperature-RT). After incubation with ×1 red blood cells lysis buffer (6 min; Biolegend, Cat. No. 420301), cells were resuspended in 5 ml of cold 1X PBS and centrifuged (800 × g, 5 min, RT). The resultant cell pellet was resuspended and plated in T25 flasks (Falcon, Cat. No. 353109) using basal preadipocyte medium containing Dulbecco’s modified Eagle’s medium F12 50:50 (Gibco, Cat. No. 11320-033), 10% fetal bovine serum (Gibco, Cat. No. 16140071), 1% (v/v) antibiotic-antimycotic (Gibco, Cat. No. 15240-062), 100 μmol/L of ascorbic acid (Sigma-Aldrich, Cat. No. A4544-25G), 33 μmol/L of biotin (Sigma-Aldrich, Cat. No. B4639-500 mg), and 20 mmol/L of HEPES 10M (pH = 7.3, ThermoScientific, Cat. No. J16924.K2) and incubated at 37°C in a humidified atmosphere with 5% CO_2_ with media replacement every 48 h. Preadipocytes were obtained by outgrowth of plastic adherent cells from the SVF cells after two serial passages in culture flasks. If performing flow cytometry in the whole SVF, resultant cells were counted using 0.4% trypan blue stain (Gibco, Cat. No. 15250-061) and an automated cell counter (Countess 3, Life Technologies Inc., Carlsbad, CA, United States), then resuspended at 1 × 10^6^ cells/ml in Fluorescence-Activated Cell Sorting (FACS) buffer containing ×1 PBS with 0.1% sodium azide (NaN_3_; Sigma-Aldrich, Cat. No. S2002-25G) and 2% fetal bovine serum (FBS, Gibco, Cat. No. 16140071).

### Adipocyte differentiation

*In vitro* cultured SAT and VAT preadipocytes were plated in distinct cell culture vessels (6-, 8-, 12-, or 24-well plates) and allowed to proliferate to confluency in basal preadipocyte medium. Adipogenic induction was performed using basal medium supplemented with 5 μmol/L of troglitazone (AdipoGen Life Sciences, Cat. No. AG-CR1-3565-M005), 0.5 mmol/L of 2 isobutyl-1-methylaxanthine (IBMX; AdipoGen Life Sciences, Cat. No. AG-CR1-3512-G001) and the following reagents from Sigma-Aldrich: 5 μg/ml of insulin (Cat. No. 10516-5ML), 10 mM acetate (Cat. No. 3863-50ML), and 1 μmol/L of dexamethasone (Cat. No. D2915-100MG). After the first 48 h, IBMX and dexamethasone were removed from the medium and cells allowed to differentiate for additional 8 days (10 days total).

### Bulk RNA sequencing and data analysis

Total RNA from adipose tissue was extracted using the RNeasy Lipid Tissue Mini kit (Qiagen, Cat. No. 74804) and RNA quality was determined using RNA Screen Tape (Agilent). Samples with RNA integrity number (RIN) greater than 7 were used in the subsequent steps. Messenger RNA purification, RNA fragmentation, double stranded cDNA, and adaptor ligation were generated using llumina Stranded mRNA Prep kit according to the manufacture’s protocol (Illumina, Cat. No. 20040534). PCR enriched libraries were quantified using the Quant-iT PicoGreen™ dsDNA Assay Kit (Invitrogen, Cat. No. P11496) and equimolar indexed libraries were pooled. Pooled libraries were checked using the Agilent Tapestation 2200 and quantified by qPCR. The libraries were then diluted to 250 p.m. and spiked with 1% phiX libraries (Illumina control). The transcriptome sequencing was performed on the barcoded stranded RNA-Seq libraries using Illumina NovaSeq 6000 flow cell, paired-end reads (2 × 50 bp) targeting at least 30 million reads per sample. FASTQ reads were trimmed for quality and adapters with TrimGalore 0.4.3 and mapped to bovine genome ARS-UCD1.2 with STAR-2.7.2a (Dobin et al., 2012) in the two-pass mode, quantMode GeneCounts including the following specifications (--outSAMstrandField intronMotif --outFilterIntronMotifs RemoveNoncanonicalUnannotated --alignEndsType Local --chimOutType WithinBAM --twopassMode Basic --twopass1readsN -1). Annotation was performed using Ensembl v106. Normalization of expression values was performed using gene length corrected trimmed mean of M-values (GeTMM) (Smid et al., 2018). Depot-specific genes (as shown in Figure 2F) were selected based on the expression in SAT or VAT samples exclusively. Differentially expressed genes were identified by One between VAT and SAT samples based upon a robust Benjamini–Hochberg corrected false discovery rate (FDR) *p value* < 0.05 (JMP 14 Pro). We used Ingenuity Pathway Analysis software (IPA, 2018) (Krämer et al., 2014) to identify activated and inhibited signaling pathways comparing VAT *versus* SAT using DEGs. The analysis output provided a–log *p value*, Z-scores, and molecules/genes for each pathway. Z-scores were considered significant if they had *p value* < 0.05 and activation Z-score >2 (activated) or < −2 (inhibited) (Menarim et al., 2021).

### Flow cytometry

We characterized the frequency of major types of cells present in SAT and VAT samples observed in the snRNA-seq analysis using flow cytometry (Attune NxT Flow Cytometer; Invitrogen, Waltham, MA, United States). Briefly, we collected VAT and SAT from an independent cohort of 10 Holstein dairy cows randomly selected at a local abattoir, and tissue was collected and processed as described above. After the SAT and VAT dissociation, cells were collected in FACS buffer followed by immunostaining with the following antibodies at 4°C for 30 min: 1.5 µg of FITC anti-bovine CD31 (ThermoScientific, Cat. No. MA1-80360), 10 µL of PE anti-bovine CD45 (ThermoScientific, Cat. No. MA1-81458), and 2 µg of unconjugated anti-human mesothelin antibody (ThermoScientific, Cat. No. PA5-79697). Primary antibodies were added to one million cells in 100 µL staining volume. For mesothelin (MSLN; ME marker) staining, 5 µg of goat anti-rabbit IgG (H + L) Alexa Fluor™ 647 (ThermoScientific, Cat. No. A-21244) was used as secondary antibody and incubated at 4°C for 15 min. Adipose stem and progenitor cells (ASPC) were defined as CD31^−^CD45^−^; EC as CD31^+^CD45^−^ and general immune cells as CD31^−^CD45^+^. Percentage of mesothelin positive cells were calculated based on total VAT or SAT SVF cells, ASPCs (CD31^−^CD45^−^), and CD31 positive or negative cells (as shown in Figure 4G) due to the uncertainty whether these cells are adipocyte progenitors or an independent pool of cells. (Chau et al., 2014; Gupta and Gupta, 2015; Westcott et al., 2021). Remaining cell populations were quantified as percentage of total cells. Controls included unstained cells and cells with single stains for each antibody. Statistical analysis was performed using the PROC MIXED of SAS (version 9.4; SAS Institute Inc., Cary, NC) with depot as fixed effect, and cow within depot as a random effect. Main effects terms were considered significant when *p value* ≤ 0.05 and tendencies when *p value* < 0.10.

### Quantitative real time PCR

*In vitro*-cultured VAT and SAT preadipocytes and adipocytes (at 0 and 10 days of differentiation, respectively) were collected in RNA lysis buffer (RLT buffer, Qiagen, Cat. No. 1015762) after Two subsequent washes with cold 1X PBS. RNA was isolated with RNeasy Mini Kit (Qiagen, Cat. No. 74104) and its concentration and integrity were evaluated in a Take3 plate (Cytation5 multi-mode reader, Biotek, Santa Clara, CA, United States). cDNA synthesis was performed with SuperScript IV VILO Master Mix (Invitrogen, Cat. No. 11756050) in a MiniAmpPlus thermal cycler (Applied Biosystems, Waltham, MA, United States; Cat. No. A37835). qRT-PCR was conducted with TaqMan^®^ gene expression assays and reagents (Life Technologies Inc., Carlsbad, CA, United States) in a QuantStudio 6 Pro (Applied Biosystems, Waltham, MA, United States; Cat. No. A43180). TaqMan assays used were *PPARG* (Bt03217547_m1), *MSLN* (Bt03263572_m1), *ADIPOQ* (Bt03292341_m1), *UPK3B* (Bt03218076_m1), *EIF3K* (Bt03226565_m1), *LUM* (Bt03211921_m1), *B2M* (Bt03251628_m1), and *WT1* (custom preparation). Data are presented as fold changes in mRNA expression calculated from least squares means differences according to the formula 2^−∆∆Ct^ (CT = cycle threshold), where ∆Ct=Ct target gene – Ct house keeping genes and ∆∆Ct=∆Ct target sample−∆Ct calibrator sample . Housekeeping genes were selected based on previous dairy cows adipose tissue studies (Strieder-Barboza et al., 2017; Strieder-Barboza and Contreras, 2019). Preadipocytes and SAT were used as calibrator samples for the differentiation and depot effects, respectively. For reporting, expression data were normalized to the arithmetic mean of the two housekeeping genes (*EIF3K* and *B2M*). Statistical analysis was performed on the ∆Ct values as described previously (Steibel et al., 2009), using the PROC MIXED of SAS (version 9.4; SAS Institute Inc., Cary, NC) with day of differentiation and depot as fixed effects, and cow within depot as a random effect. Main effects terms were considered significant when *p value* ≤ 0.05 and tendencies when *p value* < 0.10.

### Immunofluorescence microscopy

Immunofluorescence was performed with whole AT tissue and cultured preadipocytes and adipocytes. VAT and SAT preadipocytes and adipocytes (at 0 and 10 days of differentiation, respectively) cultured in glass bottom chamber-slides (Thermo Scientific, Nunc™ Lab-Tek™ II Chambered Coverglass, Cat. No. 155409) were fixed in 4% paraformaldehyde (PFA, Alfa Aesar, Cat. No. 43368) for 10 min (room temperature-RT, in the dark), rinsed three times with 1X PBS and permeabilized with 0.25% Triton X-100 (Sigma-Aldrich, Cat. No. T8787-100ML) for 15 min at RT. After rinsing twice with 1X PBS, cells were blocked for 1 h with 2% BSA-PBS at RT, followed by overnight incubation (4°C, gentle shaking) with primary antibodies. For preadipocytes, cells were stained for lumican (LUM; Invitrogen, Cat. No. MA5-34828; 1:200 antibody: 0.2% BSA-PBS) and Wilms tumor protein (WT1, Invitrogen; Cat. No. MA5-38660; 1:500 antibody: 0.2% BSA-PBS). Adipocytes were stained for adiponectin (ADIPOQ; Invitrogen, Cat. No. MA1-054; 2 μg/ml) and leptin (LEP, Bioss, Cat. No. BS-0409R; 1:200 antibody: 0.2% BSA-PBS). Following three consecutive washes with ×1 PBS, cells were co-incubated with host-specific secondary antibodies [Invitrogen, Alexa Fluor™ 488 donkey anti-rabbit IgG (Cat. No. A-21206) and Alexa Fluor™ 568 goat anti-mouse IgG (Cat. No. A-11031)] at 2 μg/ml in 0.2% BSA-PBS for 1 h at RT. Cells were subsequentially washed three times with 1X PBS and stained with DAPI (1 μg/ml, ThermoScientific, Cat. No. 62248) for 10 min at RT. Finally, cells were washed twice with 1X PBS and imaged in a Cytation5 multi-mode reader (Biotek, Santa Clara, CA, United States). For whole AT staining, samples were fixed for 20 min in 4% PFA (RT, dark room) and blocked in 1X PBS with 5% BSA + 0.1% Triton X-100 60 min RT. After Three consecutive washes with 1XPBS, antibody incubations were performed, and samples were imaged as described above. Negative controls included samples stained with only the secondary antibody. The same imaging settings were used to image controls and samples.

## Results

### Adipose tissue from dairy cows is highly heterogeneous and varies across depots

**FIGURE 1:**
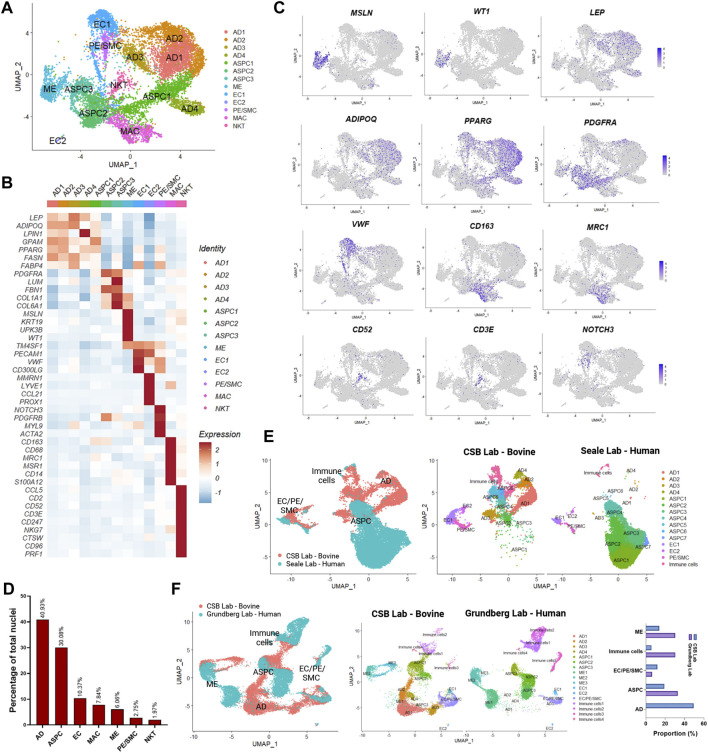
Single-nuclei RNA sequencing (snRNA-seq) analysis in subcutaneous (SAT) and visceral (VAT) adipose tissue samples from three dairy cows (n = 3). **(A)** UMAP plot of nuclei subpopulations in combined VAT and SAT samples from dairy cows. Cell populations were classified as mature adipocytes (AD), adipose steam and progenitor cells (ASPC), endothelial cells (EC), mesothelial cells (ME), pericytes and smooth muscle cells (PC/SMC), macrophages/monocytes (MAC), and natural killer and T-cells (NKT). **(B)** Heatmap of marker genes expression in cell subtypes identified by snRNA-seq. Color encodes the scaled average expression level across those cells (dark red: high expression; blue: downregulation). **(C)** UMAP plots of signature genes for ME (*MSLN* and *WT1*), AD (*ADIPOQ* and *LEP*), ASPC (*PPARG* and *PDGFRA*), EC (*VWF*), MAC (*CD163* and *MRC1*), NKT (*CD52* and *CD3E*), PC/SMC (*NOTCH3*). Purple dots represent individual nucleus expressing the respective marker. **(D)** Percentages of nuclei per cell type across SAT and VAT. **(E)** Integrated analysis of cell types identified by our database (CSB Lab-Bovine) compared with human scRNAseq data generated by Merrick et al. (2019) (Seale Lab - Human). **(F)** Integrated analysis of cell types identified by our database (CSB Lab-Bovine) compared with human scRNAseq data generated by Vijay et al. (2020) (Grundberg Lab – Human). Bar graph represents the comparison between the proportions of distinct cell types found in each study.

We generated snRNA-seq data from abdominal SAT and omental VAT samples derived from Holstein dairy cows (Supplementary Figures S1A–G). Across six matched SAT and VAT samples, 13 clusters (Figure 1A) were identified among 11,271 nuclei, in which 7,018 pertained to SAT and 4,253 to VAT. Manual annotation via expression of signature genes defined cell types (Supplementary Table S1), which consisted of mature adipocytes (AD; *ADIPOQ, LEP*), adipose stem and progenitor cells (ASPC; *PDGFRA, PPARG*), endothelial cells (EC; *VWF, PECAM1*), macrophages/monocytes (MAC; *CD163, MRC1, CD14*), natural killer and T-cells (NKT; *CD52, CD3E*), mesothelial cells (ME; *WT1, MSLN*), and pericytes/smooth muscle cells (PE/SMC; *NOTCH3, MYL9*) (Figures 1B,C). Across SAT and VAT, AD was the most abundant cell type, followed by ASPC, EC, MAC, ME, PE/SMC, and NKT (Figure 1D).

We compared our dataset from dairy cow AT with recently published scRNA-seq datasets from human SAT and VAT (Figures 1E,F) (Merrick et al., 2019; Vijay et al., 2020). We observed similarities in the identification of cell types, such as ASPC, EC, ME, PE/SMC, and immune cells with both datasets. As expected from scRNA-seq performed with AT SVF, mature adipocytes were practically absent. Human SAT from the Merrick et al. (2019) dataset revealed a strong and diverse presence of ASPC populations, while immune cells, EC, and PE/SMC were proportionally less abundant in comparison to our dairy cow SAT data (Figure 1E). Across SAT and VAT, human AT from Vijay et al. (2020) dataset presented greater abundance of ME and immune cells than our results from dairy cows (Figure 1F) and may be related to AT species-specific characteristics or distinct methods to dissociate cells vs. nuclei from AT samples.

### Adipose tissue of dairy cows has depot-specific cell subpopulations and characteristics

**FIGURE 2:**
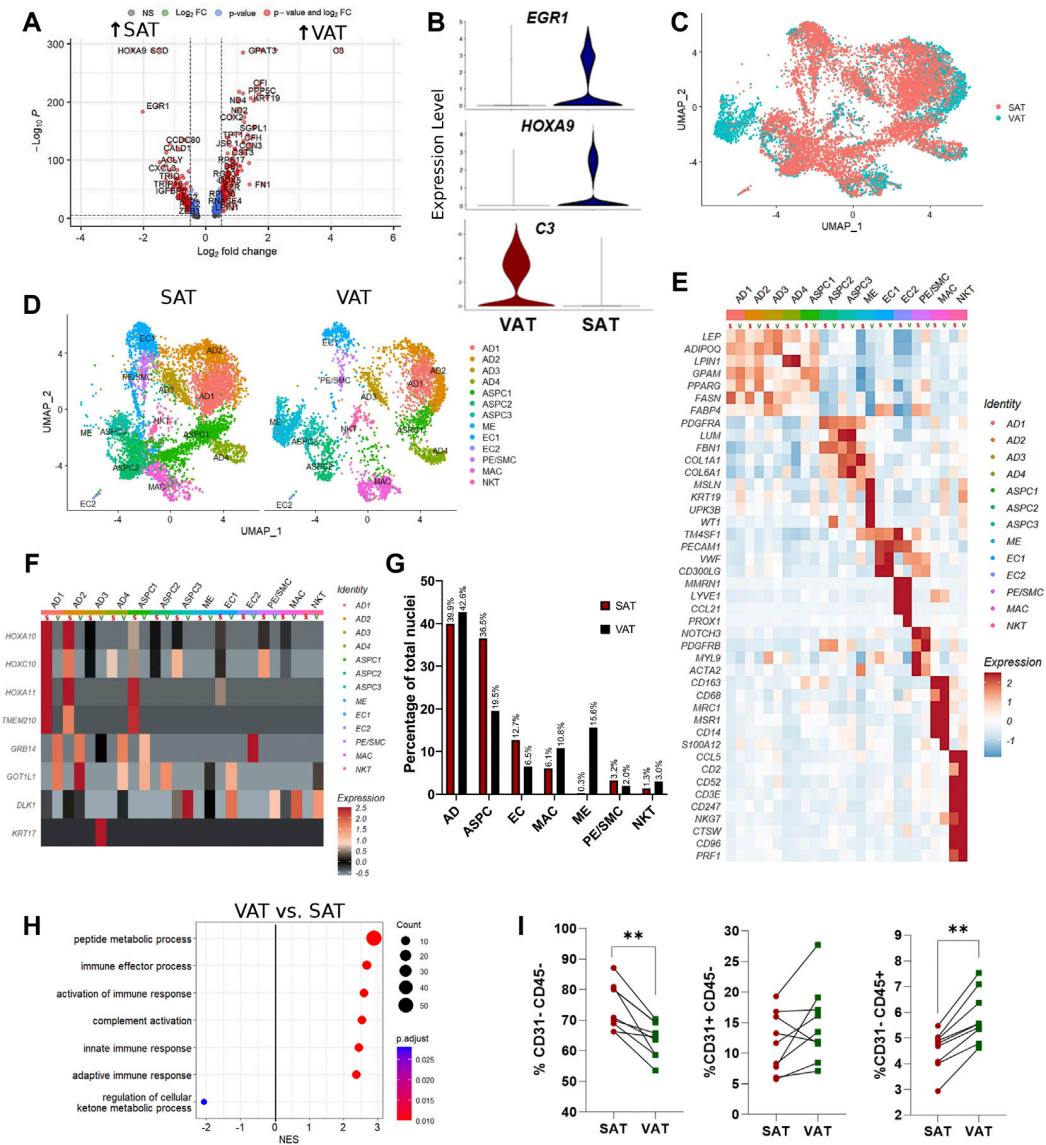
Depot-specific differences revealed by snRNA-seq and bulk-RNAseq in adipose tissues of dairy cows. **(A)** Volcano plot obtained from single-nuclei RNA sequencing (snRNA-seq) analysis comparing the overall expression of distinct genes between subcutaneous (SAT) and visceral (VAT) adipose tissue samples from dairy cows (n = 3). **(B)** Violin plots of genes with the highest contrast between depots from snRNA-seq analysis. The *y* axis indicates log-transformed expression values, and the width indicates the number of cells expressing the gene. **(C)** Overlapping VAT (blue) and SAT (red) UMAP plots highlighting depot-specific differences on abundance of specific cell types. **(D)** UMAP plots of VAT and SAT containing all identified cell types. **(E)** Heatmap of marker genes expression in cell subtypes identified according to depot (S = SAT; V = VAT). Color encodes the scaled average expression level across those cells (high expression; blue: downregulation). **(F)** Heatmap of cell type specific expression patterns of the top differentially expressed genes (DEGs) in VAT and SAT depots (S = SAT; V = VAT) identified by bulk-RNAseq. Color encodes the scaled average expression level across those cells (high expression; grey: downregulation). **(G)** Comparison of nuclei proportions per cell type between SAT and VAT. **(H)** Pathway analysis (GSEA) of DEG between VAT and SAT. Positive normalized enrichment score (NES) represents pathway activation on VAT, while a negative NES represents pathway suppression on VAT when compared with SAT. **(I)** Flow cytometry analysis of percentage of ASPC (CD31^−^CD45^−^), EC (CD31^+^CD45^−^), and immune cells (CD31^−^CD45^+^) in SAT and VAT SVF in an independent cohort of dairy cows (n = 10).

Single-nuclei RNA sequencing analysis of SAT and VAT revealed more than 300 differentially expressed genes (DEGs) between the depots (Figures 2A,B). There was a marked increase in the expression of components of the complement system on VAT, especially *C3* – the gene with the highest contrast between VAT and SAT (Log_2_FoldChange = 4.26) (Figures 2A,B). In contrast, the gene expression profile of SAT revealed an increased expression of *EGR1*, genes of the FOS family (*FOS* and *FOSB*), fatty acid synthesis (*FASN, ACLY,* and *SCD*), and Hox genes (*HOXA9, HOXD4, HOXC6,* and *HOXA6*) (Supplementary Table S5). All main cell types, including AD, ASPC, EC, PE/SMC, MAC, and NKT were present in both depots, except for ME, which was a VAT-specific cell subtype, with little to no expression in SAT (Figures 2C,D). When comparing average gene expression of cell type signature genes between VAT and SAT (Figure 2E), we identified several DEGs that were depot-specific, such as the increased expression of *FASN* in SAT AD subpopulations compared with VAT, the increased expression of ME-markers *WT1, UPK3B,* and *MSLN* in VAT compared with SAT, and the decreased expression of *CD68* and S*100A12* in SAT MAC compared with VAT. Taken together, these data suggest depot-specific differences in AT cell types, which may reflect distinct metabolic and immune functions for VAT and SAT.

Evaluation of the top SAT and VAT DEG from our bulk RNA-Seq analysis support cell type-specific gene expression (Figure 2F). SAT-specific *HOXA10, HOXC10, HOXA11* and *TMEM210* are specially enriched in SAT AD and ASPC. *GRB14* and *GOT1L1* are unique to VAT ADs and ASPCs, while *KRT17* and *DLK1* are expressed only in VAT AD and ASPC, respectively. These findings validate our snRNA-seq methodology in identifying depot-specific cell subtypes in which AT bulk RNA-seq DEGs are expressed.

Mature adipocytes (AD), defined as nuclei expressing *ADIPOQ* and*/*or *LEP,* were the most frequent cell type detected in both VAT and SAT, corresponding to approximately 40% of total nuclei (Figure 2G). Abundance of EC and ASPC was increased in SAT compared to VAT by approximately 2-fold, suggesting increased angiogenic and adipogenic capacity, respectively, in SAT compared with VAT in dairy cows. Notably, VAT has higher proportions of both MAC and NKT compared with SAT (Figure 2G), which agrees with the enrichment in pathways of complement activation and immune responses in VAT compared with SAT (Figure 2H). Enrichment analysis also implied a decreased capability of VAT to regulate cellular ketone metabolic process, thus indicating the involvement of VAT dysfunction in ketosis pathogenesis in dairy cows. We validated the snRNA-Seq results using flow cytometry analysis to quantify proportions of bulk ASPC and immune cells in VAT and SAT samples obtained from an independent cohort of dairy cows (Figure 2I).

### Adipose stem and progenitor cell subpopulations have adipogenic and fibro-adipogenic profiles

**FIGURE 3:**
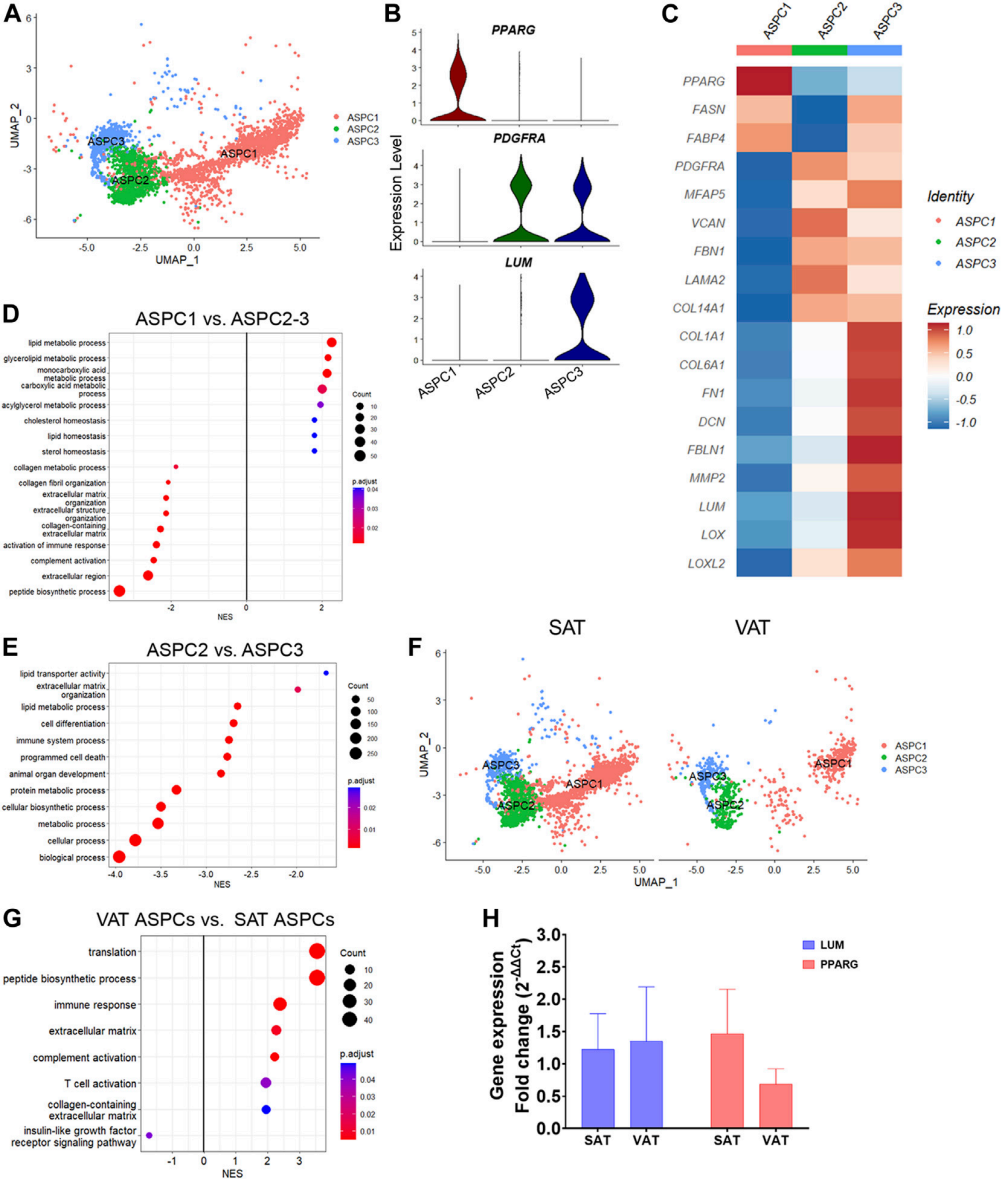
Transcriptional diversity of adipose stem and progenitor cells (ASPC) in visceral (VAT) and subcutaneous (SAT) adipose tissue of dairy cows. **(A)** UMAP plot of ASPCs across SAT and VAT. **(B)** Violin plots of ASPC markers *PPARG, PDGFRA*, and *LUM* in the distinct ASPC subtypes. The *y* axis indicates log-transformed expression values, and the width indicates the number of cells expressing the gene. **(C)** Heatmap of ASPC markers genes in the distinct identified subtypes. Color encodes the scaled average expression level across those cells (high expression; blue: downregulation). **(D)** Pathway analysis (GSEA) of differentially expressed genes (DEG) between adipogenic ASPC1 and FAP ASPC2/3. Positive normalized enrichment score (NES) represents pathway activation on ASPC1, while a negative NES represents pathway suppression when compared with ASPC2-3. **(E)** Pathway analysis (GSEA) of differentially expressed genes between FAP ASPC2 and FAP ASPC3. Positive normalized enrichment score (NES) represents pathway activation on ASPC2, while a negative NES represents pathway suppression when compared with ASPC3. **(F)** UMAP plots of VAT and SAT ASPCs. **(G)** Pathway analysis (GSEA) of differentially expressed genes between overall VAT and SAT ASPC types. Positive normalized enrichment score (NES) represents pathway activation on VAT ASPCs, while a negative NES represents pathway suppression when compared with SAT ASPCs. **(H)** Comparison of adipocyte relative to preadipocyte gene expression fold change of *LUM* and *PPARG* between SAT and VAT obtained from an independent cohort of dairy cows (n = 4). For gene expression fold change calculations, adipocyte *PPARG* and *LUM* expression were calibrated by preadipocyte expression in matched SAT and VAT samples from each cow.

We identified three distinct subtypes of adipose stem and progenitor cells (ASPC1-3, Figure 3A; Supplementary Table S6). ASPC1 showed greater expression of *PPARG* (Figures 3B,C), a master regulator of lipid biosynthesis and adipocyte differentiation (Schadinger et al., 2005; Guo et al., 2019), as well as *SLC1A3, LIPE*, *GPAM* and *LMO4*, suggesting that these cells are committed adipocyte precursors (Hepler et al., 2018). In contrast, ASPC2 and ASPC3 showed increased expression of *PDGFRA* (Figures 3B,C), a known marker of fibro-adipogenic progenitor cells (FAP), which have the capability to differentiate into adipocytes or activated fibroblasts (Contreras et al., 2021; Dohmen et al., 2022). Compared with ASPC1, the *PDGFRA* ^*+*^ ASPC2 and ASPC3 FAPs had greater expression of extracellular matrix (ECM) genes, such as *FBN1, FN1, LAMA2, COL14A1,* and *MFAP5* (Figure 3C). Interestingly, ASPC3 was uniquely enriched for *COL1A1, COL6A1, FN1*, *LOX*, and *LUM,* which are fibrosis markers recently associated with a specific subset of *PDGFRA* ^*+*^ progenitor cells in murine model of obesity (Marcelin et al., 2017).

Enrichment analysis of adipogenic ASPC1 vs. ASPC2 and ASPC3 FAP subtypes (Figure 3D; Supplementary Table S3) revealed the activation of pathways related to lipid metabolism (e.g., lipid metabolic process, glycerolipid metabolic process, acylglycerol metabolic process, and cholesterol homeostasis) in ASPC1, while pathways associated to ECM, collagen-containing ECM, complement activation, and immune response were enriched in FAP ASPC2 and ASPC3 (Figure 3D). Next, we investigated differences between the two FAP ASPC subtypes detected in both SAT and VAT of dairy cows. Analysis of ASPC2 DEG revealed a greater expression of “classic” FAP markers, such as components of ADAM (metalloprotease—disintegrin) family, *BMP1, EBF1, FGFR1*, and *TGFB* receptors (that may participate into FAP fibrogenic differentiation). In contrast, ASPC3 had *COL1A1, COL1A2, COL6A1, FN1, DCN, FBLN1, MMP2, LUM, OGN, SPARC,* and *LOX,* known markers of tissue fibrosis (Sun et al., 2013), among the most upregulated genes. Several of these markers have been consistently associated with AT fibrosis, decreased adipogenic capacity, and dysfunction in human and mice models. Additionally, ASPC3 had increased expression of pro-inflammatory markers, e.g., *CCL2, CXCL3, C3, C1S,* and *PTGS2,* similar to the previously reported fibro-inflammatory progenitors (‘FIPs’) in mice AT, which exhibited a pro-fibrogenic/pro-inflammatory profile (Hepler et al., 2018). Consistently, enrichment analysis comparing “classic-FAP” ASPC2 vs. “fibrogenic FAP” ASPC3 revealed an upregulation of pathways associated with ECM organization, immune system process, cell differentiation, and programmed cell death (Figure 3E; Supplementary Table S3). Overall, these findings suggest that ASPC3 has a pro-inflammatory and profibrogenic profile that may drive AT fibrosis and negatively affect adipogenesis.

There was a greater proportion of ASPC in SAT than in VAT (Figure 3F), which was confirmed by flow cytometry analysis of SAT and VAT SVF (Figure 1I). Notably, the proportion of adipogenic ASPC1 was decreased by 10% in VAT compared with SAT, implying a decreased adipogenic capacity of VAT compared to SAT. Enrichment analysis of VAT ASPCs vs. SAT ASPCs (Supplementary Table S2) revealed an activation of immune response, complement activation, and ECM pathways, and a suppression of insulin-like growth factor receptor signaling pathway (Figure 3G), suggesting a pro-inflammatory potential of VAT ASPCs. Moreover, overall gene expression comparison between depots (Supplementary Table S7) revealed an upregulation of many complement system genes, such as *C3, C4a, C1QC, C1S, C1R,* and *SERPING1*, involved in the regulation of the complement cascade (Gorelik et al., 2017; Lubbers et al., 2020), as well as pro-fibrotic markers, *LUM, FN1,* and *FBLN1* in VAT ASPCs. In contrast, *FASN, SCD*, *IGFBP5,* and *IGFBP3* were upregulated in SAT ASPCs. We observed no differences on *PPARG* and *LUM* gene expression between SAT and VAT in *in vitro* cultivated using qRT-PCR analysis (Figure 3H; n = 4).

### Mesothelial cells are a VAT-specific cell subtype

**FIGURE 4:**
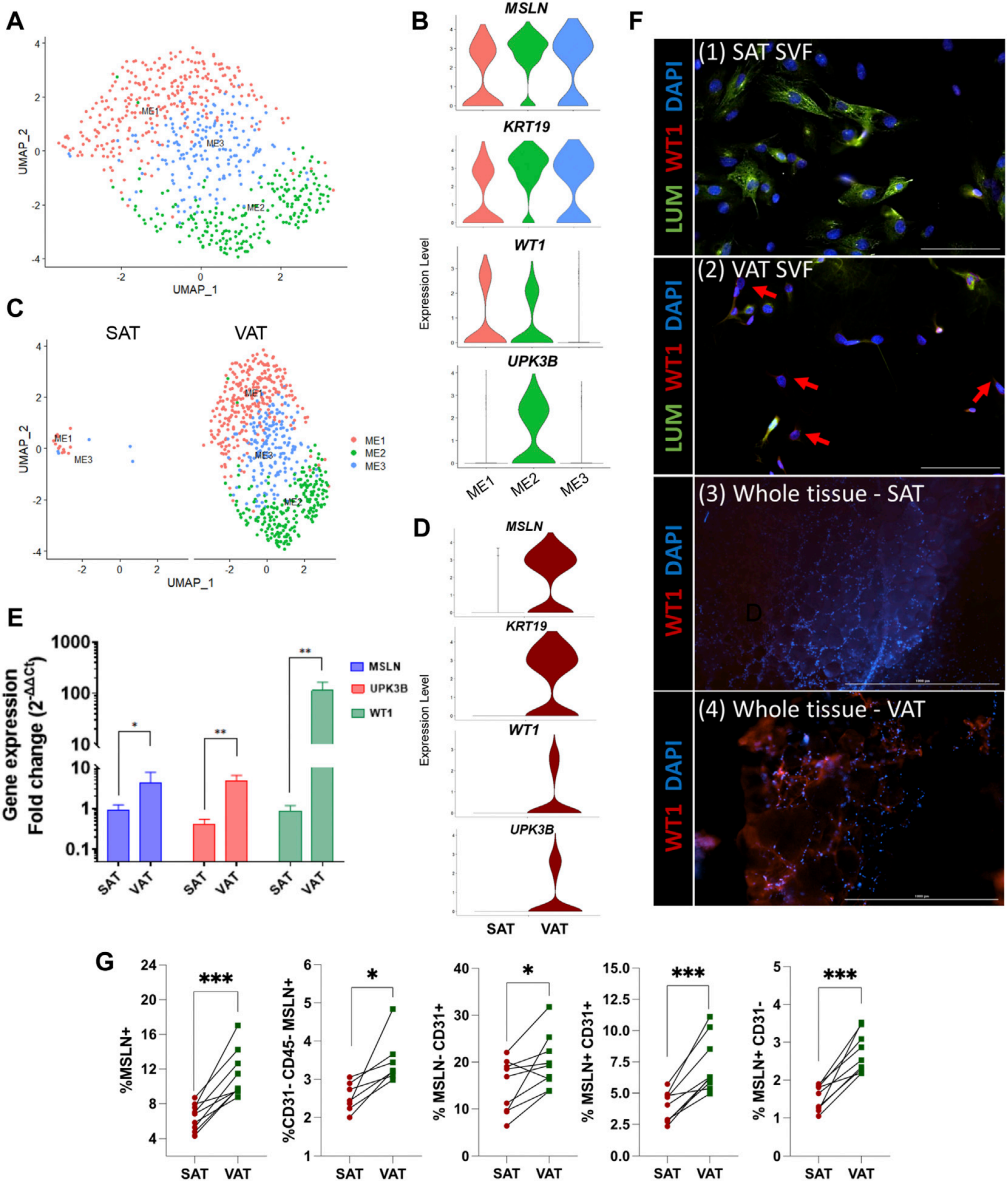
Mesothelial cells are a VAT-specific cell type. **(A)** UMAP plot of ME across visceral (VAT) and subcutaneous (SAT) adipose tissue of dairy cows (n = 3) obtained through snRNA-seq analysis. **(B)** Violin plots of ME marker genes *MSLN, KRT19, WT1* and *UPK3B* in ME subtypes. The *y* axis indicates log-transformed expression values, and the width indicates the number of cells expressing the gene. **(C)** UMAP plots of VAT and SAT ME. **(D)** Violin plots of ME marker genes in SAT and VAT. The *y* axis indicates log-transformed expression values, and the width indicates the number of cells expressing the gene. **(E)** Comparison of adipocyte relative to preadipocyte gene expression fold change of ME marker genes (*MSLN, UPK3B,* and *WT1*) between SAT and VAT (n = 4). For gene expression fold change calculations, adipocyte *MSLN, UPK3B,* and *WT1* expression were calibrated by preadipocyte expression in matched SAT and VAT samples from each cow. **(F)** Immunofluorescence imaging of (1) SAT and (2) VAT SVF stained with LUM (green), WT1 (red), and DAPI (blue) (×20 objective, 100 µm scale bars), and (3) SAT and (4) VAT whole tissue (×4 objective, 1,000 µm scale bars) stained with WT1 (red), and DAPI (blue). Red arrows highlight WT1+ cells. **(G)** Flow cytometry analysis of SAT and VAT SVF from an independent cohort of dairy cows (n = 10) showing percentages of (from left to right): Total ME cells (MSLN+); ME cells (MSLN+) in ASPC (CD31^−^CD45^−^) population; CD31^+^ cells that do not express MSLN; CD31^+^ cells that express MSLN; and ME cells (MSLN+) that do not express CD31. *p value ≤ 0.05 and; ***p value ≤ 0.0001 using paired *t*-test.

Mesothelial cells were annotated based on the expression of the signature marker genes *MSLN*, *KRT19*, *WT1*, and *UPK3B*. Subclustering of ME resolved three major subtypes (Figure 4A). When contrasting the distinct ME subpopulations, we observed a unique pattern of *WT1* and *UPK3B* expression. While *MSLN* and *KRT19* were expressed in all ME subtypes, ME1 was *WT1* ^*+*^ *UPK3B* ^*−*^, ME2 was *WT1* ^*+*^ *UPK3B* ^*+,*^ and ME3 W*T1* ^*-*^ *UPK3B* ^*−*^ (Figure 4B). Interestingly, ME2 was enriched for genes associated with inflammation, such as *C3, CFB, C1S*, and *CD99*, as well as genes related to adipogenesis (*CD34, IGF2*) and fibrosis (*CD9, SPARC, COL8A1*) (Marcelin et al., 2017). The potential roles of ME on AT inflammation, adipogenesis, and fibrosis and whether their distinct transcriptional profiles translate into functional differences among ME subtypes remain to be established. We observed strong depot differences: ME were practically absent in SAT (0.3% of total nuclei), while ME represented around one sixth of total population of VAT cells (15.6%; Figure 4C). This was also confirmed by the marked upregulation of *MSLN* and *KRT19,* and to a lesser extent *WT1* and *UPK3B* in VAT vs. SAT in an independent cohort of dairy cows (Figure 4D). These results agree with previous snRNA-seq and scRNA-seq studies in mouse and human models, which indicate a specific expression of *MSLN* and other mesothelial markers in VAT (Vijay et al., 2020; Emont et al., 2022). We validated our snRNA-seq results by qRT-PCR, flow cytometry, and immunofluorescence: qRT-PCR showed greater mRNA expression of the ME markers *MSLN*, *WT1* and *UPKB3* in VAT compared to SAT (Figure 4E; n = 4). Accordingly, whole tissue immune-stained with WT1 confirmed the absence of WT1 expression in SAT, but abundant expression in VAT (Figure 4F). Using flow cytometry, we confirmed a greater proportion of MSLN positive cells in SVF from VAT compared to SAT (Figure 4G). Finally, immunofluorescence imaging of SVF revealed few cells expressing WT1 in VAT, but not in SAT (Figure 4F); additionally, we observed that a few VAT cells may co-express WT1 and LUM, which was upregulated in FAP ASPCs in our snRNA-seq analysis.

### Mature adipocytes are transcriptionally distinct and mimic ASPC profiles

**FIGURE 5:**
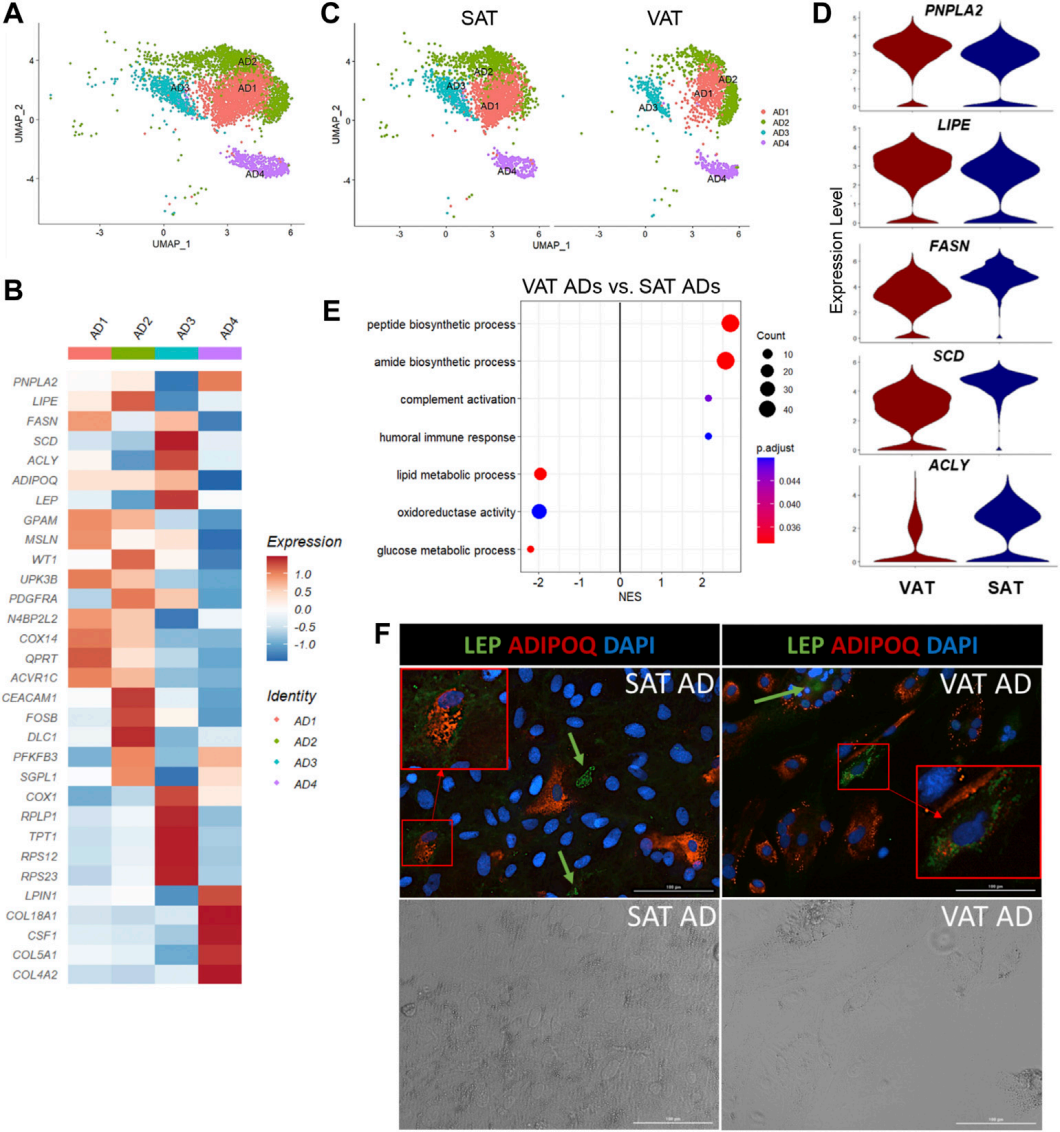
Mature adipocytes are not a homogeneous cell type in adipose tissue of dairy cows. **(A)** UMAP plot of adipocytes (AD) across visceral (VAT) and subcutaneous (SAT) adipose tissue of dairy cows (n = 3) obtained through snRNA-seq analysis. **(B)** Heatmap of gene expression in AD subtypes. Color encodes the scaled average expression level across those cells (dark red: high; blue: downregulation). **(C)** UMAP plots of VAT and SAT AD subtypes**. (D)** Violin plots of different lipid metabolism genes between SAT and VAT. *PNPLA2* and *LIPE* expression was higher in VAT, while *FASN, SCD*, and *ACLY* was higher in SAT. The *y* axis indicates log-transformed expression values, and the width indicates the number of cells expressing the gene. **(E)** Pathway analysis (GSEA) of differentially expressed genes between VAT and SAT AD. Positive normalized enrichment score (NES) represents pathway activation on VAT ADs, while a negative NES represents pathway suppression when compared with SAT ADs. **(F)** Immunofluorescence imaging of SAT and VAT adipocytes after 10 days of *in vitro* differentiation stained with LEP (green), ADIPOQ (red), and DAPI (blue) in upper panel. Bottom panel includes phase contrast images of the same imaging field. Green arrows highlight LEP + cells. Red squares highlight zoomed in LEP + ADIPOQ + cells. All images were obtained with a ×20 objective. Scale bars: 100 µm.

Our snRNA-seq approach allowed us to recollect data about distinct subtypes of mature adipocytes, which are usually excluded from scRNA-seq due to the incompatibility of adipocyte size with microfluidics and/or the absence of mature adipocytes in studies using AT SVF cells. Our analysis resolved four different populations of mature adipocytes (AD1-4, Figure 5A) annotated based on the expression of *ADIPOQ* and/or *LEP*. While AD1 and AD3 were characterized by the increased expression of both *ADIPOQ* and *LEP* (*ADIPOQ* ^*+*^ *LEP* ^*+*^)*,* AD2 and AD4 were characterized by the selective expression of *ADIPOQ* (*ADIPOQ* ^*+*^) or LEP (*LEP* ^*+*^)*,* respectively (Figure 5B). While AD4 had the lowest average expression of *LEP* and downregulation of *ADIPOQ*, *LPIN1* was one of the most upregulated genes in AD4. *LPIN1* is a reciprocal regulator of triglyceride synthesis and hydrolysis in adipocytes (Mitra et al., 2013), therefore, a marker of adipocytes (Supplementary Table S8). Immunofluorescence imaging of adipocytes stained with ADIPOQ and LEP corroborate with our snRNA-seq findings, in which we observed that adipocytes have a selective expression of ADIPOQ or LEP, while other cells seem to express both proteins (Figure 5F).

Based on DEG analysis between AD subtypes (Supplementary Table S8), we examined for evidence of ADs with profiles similar to adipogenic or fibro-adipogenic ASPC, or ME cells. We observed that AD1 has an adipogenic profile (Figure 5B), like ASPC1, with high expression of classical lipid synthesis regulators*.* In contrast to AD1, AD2 and AD3 have a FAP ASPC-like gene profile. For example, *PDGFRA* and ECM genes, including *DCN, FBN1, LAMA2*, and *COL1A1/A2* are upregulated in AD2, similar to our FAP ASPC3. AD3 has a FAP-like profile due to the upregulation of genes involved in adipogenesis and lipid metabolism (*ADIRF, THRSP, SCD, ACLY, FABP4, AGPAT2, APOE,* and *LPL*), as well as ECM genes, such as *CLU, VIM*, and *SPARC.* Interestingly, AD3 also had a greater expression of mitochondrial-related genes, including *ND4, ND1, COX1, COX2,* and *COX3,* when compared to the other AD subtypes, which might indicate a brown adipose tissue-like profile, or be correlated with greater adipocyte lipogenesis and mitochondrial oxidative capacity in this specific subpopulation (Kaaman et al., 2007; Lu et al., 2018). AD4 had a unique profile with high *LPIN1, CSF1,* and collagens (*COL18A1, COL5A1*, and *COL4A2*) expression, implying a FAP-like profile that does not overlap with AD2 and AD3. We did not find significant similarities between AD4 and ASPCs or ME cells. ME gene markers, such as *MSLN, KRT19, WT1*, and *UPK3B* were not differentially expressed in AD subtypes. However, AD1, AD2, and AD3 subtypes expressed at least one of these markers (Figure 5B). Since heatmaps are based on average expression, there is a possibility that only a few cells within AD1-3 subtypes expressed high ME markers, while others did not express any of them at all. Enrichment analysis revealed activation of pathways related to protein synthesis and mitochondria respiratory chain complex on AD3 when compared to other AD populations, while AD2 showed an enrichment in cell differentiation and development pathways (Supplementary Table S4). No pathways were significantly activated in AD1 or AD4.

All AD subpopulations were present in both VAT and SAT (Figure 5C) and had similar proportions within each depot. Overall, VAT ADs had increased expression of complement system genes (*C3, CFB, C1QA, C1QB*, and *C1QC*)*,* as well as *SERPING1* (Supplementary Table S9). Moreover, we observed an increased expression of *PPARG*, *LPIN1, PNPLA2,* and *LIPE* in VAT adipocytes when compared to SAT (Figure 5D). In contrast, SAT adipocytes had greater expression of *EGR1* and *de novo* fatty acid synthesis genes (*FASN, ACLY,* and *SCD*) (Figure 5D). Interestingly*,* seven heat shock protein genes (e.g., *HSPA8* and *HSPB1*) were upregulated in SAT, but not in VAT (Supplementary Table S9). No depot-differences were observed in *ADIPOQ* expression, which was confirmed *in vitro* through *ADIPOQ* mRNA quantification in adipocytes after 10 days of differentiation (*p value >* 0.10). Enrichment analysis comparing VAT vs. SAT ADs revealed a suppression of glucose and lipid metabolic process, steroid biosynthetic process, and oxidoreductase activity pathways, and an activation of pathways related to protein synthesis and humoral immune response in VAT (Figure 5E, Supplementary Table S2).

### Macrophages are the predominant immune cell type in VAT and SAT of dairy cows

**FIGURE 6:**
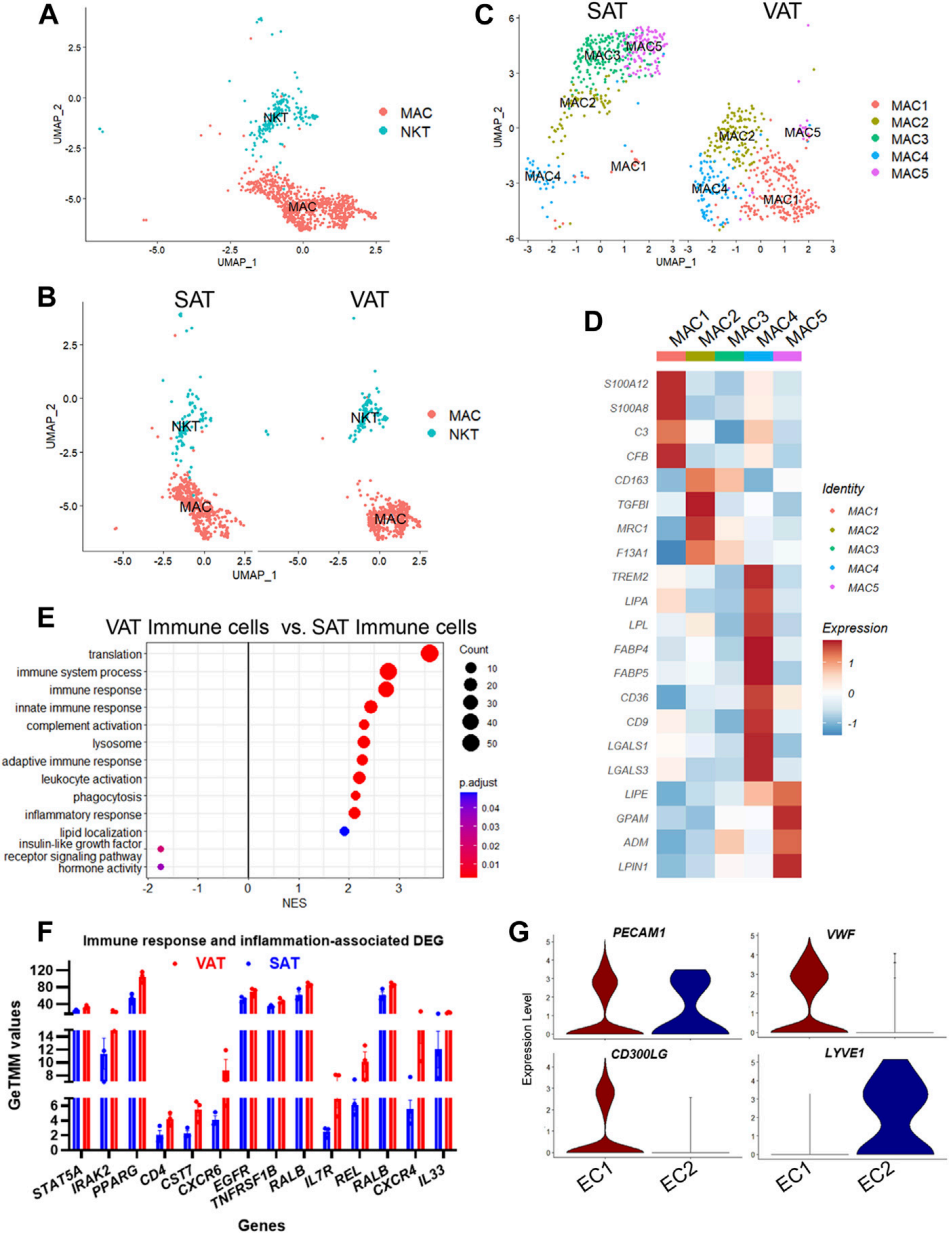
Adipose tissue of dairy cows contains distinct subpopulations of immune and endothelial cells. **(A)** UMAP plot of macrophages/monocytes (MAC) and natural killer and T-cells (NKT) across visceral (VAT) and subcutaneous (SAT) adipose tissue of dairy cows (n = 3) obtained through snRNA-seq analysis. **(B)** UMAP plots of VAT and SAT MAC and NKT subtypes. **(C)** UMAP plots of MAC subtypes in SAT and VAT. **(D)** Heatmap of MAC subtypes gene markers in VAT and SAT. Color encodes the scaled average expression level across those cells (dark red is high and blue is low). **(E)** Pathway analysis (GSEA) of differentially expressed genes between VAT and SAT immune cells. Positive normalized enrichment score (NES) represents pathway activation on VAT immune cells, while a negative NES represents pathway suppression when compared with SAT immune cells. **(F)** Expression of immune and inflammatory DEGs between VAT and SAT from bulk RNA-seq. **(G)** Violin plots of EC marker genes in EC1 (vascular EC) and EC2 (lymphatic EC). The *y* axis indicates log-transformed expression values, and the width indicates the number of cells expressing the gene.

We identified two major clusters of immune cells (MAC and NKT; Figures 6A,B), which represented 9.8% of total nuclei across SAT and VAT (Figure 2D). MAC expressed markers of both macrophages (*MRC1, MSR1, CD68,* and *CD163*) and monocytes (*S100A12* and *CD14*), while NKT had increased expression of T-cell markers, including *CCL5, CD3E, CD2, CD247,* and *CD52,* and natural killer cells, such as *NKG7* and *CTSW* (Figure 2B)*.* Across depots, mononuclear phagocytes (macrophages and monocytes) were the most abundant immune cell type (80% of immune cells nuclei), while lymphocytes (T-cells and NK cells) represented the remaining 20%. Comparison between AT depots showed that VAT has greater proportion of both NKT (3.0% vs. 1.3% of total nuclei) and MAC (10.8% vs. 6.1%) when compared to SAT (Figure 1G). NKT had an increased expression of *CCL5,* a chemokine involved in the chemotaxis of activated T cells (Murooka et al., 2008; Chan et al., 2012), with approximately 35% of NKT cells expressing *CCL5* (Supplementary Table S1), suggesting a considerable level of immune cell activation.

Sub-analysis of MAC identified five main macrophage subtypes (MAC1–5, Supplementary Table S10). All MAC subclusters were present across both VAT and SAT depots, except MAC3, present exclusively on SAT (Figure 6C). We also identified high expression of *S100A1*2 and *S100A8* in MAC1, suggesting these cells are monocytes or differentiating macrophages (Jaitin et al., 2019; Vijay et al., 2020; Strieder-Barboza et al., 2022). There was a significant upregulation of several complement and complement receptor genes in MAC1, such as *C3, CFI, CFB, CD55, CFH, CR2, C1QC, C1QB*, and *C1QA* among others, suggesting MAC1 cells involvement on complement activation during inflammation in AT. MAC2 were enriched for *CD163*, a marker for perivascular macrophage, *TGFBI, MRC1/CD206*, and *F13A1*. Both, *CD206+* and *F13A1+* macrophages, have been associated with AT dysfunction, pro-inflammatory responses, and AT remodeling in human obesity (Kaartinen et al., 2021; Muir et al., 2022). SAT-specific MAC3 was majorly characterized by *ABL1,* which’s expression regulates macrophage podosome formation, *SPTBN1, ZBTB16,* and *ADAMTSL3*. In agreement with previous studies in human AT (Jaitin et al., 2019; Vijay et al., 2020; Strieder-Barboza et al., 2022), MAC4 had a lipid associated macrophage (LAM) profile with high expression of *FABP4*, *LPL*, *CD36, FASN, CD9,* among other lipid related genes (Figure 6D, Supplementary Table S10). Abundance of MAC4 nuclei was increased by approximately 2-fold in VAT compared to SAT (20.3% vs. 10.6%) and SAT MAC4 had increased expression of lipogenic genes (*FASN, SCD*, and *ACLY*) when compared to VAT MAC4 (Supplementary Table S12). Finally, MAC5 showed an increased expression of genes related to lipid metabolism other than those associated with LAM, e.g., *LIPE, GPAM, ADM,* and *LPIN1*. Notably, *LPIN1*, has been reported as a mediator of macrophage pro-inflammatory activation and a link between lipid biosynthesis and systemic inflammatory responses (Meana et al., 2014).

A general comparison of immune cell gene expression between depots (Supplementary Table S11) revealed that VAT immune cells had greater expression of complement system genes *(C3, CFB, C1QA, C1QB,* and *C1QC*)*,* bovine major histocompatibility complex genes (*BOLA-DRA* and *BOLA-DMR*), S100 protein family genes (*S100A9, S100A10, S100A11,* and *S100A12*), and phagocytes oxidase system genes (*CYBA* and *CYBB*) compared with SAT immune cells. We observed the activation of GO pathways related to immune and inflammatory response, complement activation, phagocytosis, and leukocyte migration and mediated immunity in VAT immune cells (Figure 6E, Supplementary Table S2). In addition, bulk RNA-seq analysis revealed a greater expression of key immune response and inflammation-related DEG in VAT compared to SAT (Figure 6F; Supplementary Table S16), including *CD4, CXCR6, CXCR4, IL7R, REL, RALB,* and *IL33.* Accordingly, we observed the activation of numerous immune response and inflammation-related pathways in VAT compared to SAT (Table 1). Interestingly, several classical inflammatory genes were not differentially expressed (FDR>0.05) but were present in the transcriptome of VAT and SAT, including *IL4, CSF1, CD68, CD86, CD83, CD80, IL1B, CSF1R, STAT1, RXRG, REL, IL1A, GPR85, IL6, RXRA, FOXP3, NFKB2, RELA, IL4R, RXRB,* and *STAT6* (Supplementary Table S15). Taken together, these results highlight a greater abundance of immune cells in VAT, which may be associated with an increased inflammatory response as these cells seem to be more immunologically activated when compared to SAT immune cells.

**TABLE 1:** Immune response and inflammation-related activated pathways in VAT versus SAT samples identified by Ingenuity Pathways Analysis (IPA) from bulk-RNAseq data.

Pathway	z-score	−log (*p* value)	Detected genes
T Cell Receptor Signaling	2.50	0	*AKT1, BCL10, CBL, CD4, CHP1, FCER1G, FOS, FYB1, GRAP2, HLA-DOA, HLA, DQA1, ITGA2, ITGAL, ITGB2, JUN, LCP2, MAP2K6, PDK1, PIK3CD, PIK3CG, PIK3R2, PTPN6, RALB, REL, SKAP1, TCF7, ZAP70*
NF-κB Signaling	2.24	0	*AKT1, BCL10, BRAF, EGFR, EP300, FCER1G, GHR, IL33, MAP2K6, NTRK3, PDGFRB, PIK3CD, PIK3CG, PIK3R2, PRKCB, RALB, TNFRSF11A, TNFRSF1B, TNFSF11, ZAP70*
PKCθ Signaling in T Lymphocytes	2.18	0	*BCL10, CACNA1A, CATSPER2, CATSPER3, CD4, CHP1, FCER1G, FOS, GRAP2, HLA-DOA, HLA-DQA1, ITPR1, ITPR2, JUN, LCP2, MAP3K10, PIK3CD, PIK3CG, PIK3R2, RAC2, RALB, REL, ZAP70*
Natural Killer Cell Signaling	2.24	1.09	*AKT1, CD244, COL1A1, COL1A2, FCER1G, IL12RB2, IL2RB, ITGAL, KLRC1, LCP2, MAP3K10, PIK3CD, PIK3CG, PIK3R2, PTPN6, RAC2, RALB, REL, SYK, TNFSF10, ZAP70*
fMLP Signaling in Neutrophils	2.14	2.28	*ARPC1A, ARPC1B, ARPC3, CHP1, GNAO1, GNAQ, GNAS, GNAZ, GNB1, ITPR1, ITPR2, PIK3CD, PIK3CG, PIK3R2, PLCB4, PRKCB, RAC2, RALB, REL*
PI3K Signaling in B Lymphocytes	2.07	2.45	*AKT1, ATF6, ATF7, BCL10, BLNK, CBL, CD180, CHP1, FOS, ITPR1, ITPR2, JUN, PIK3CD, PIK3CG, PIK3R2, PLCB4, PLEKHA4, PRKCB, RALB, REL, SYK*
Phagosome Formation	2.52	3	*ADGRE1, ADGRE4, ADGRE5, ADGRF3, ADGRF5, ADGRG6, ADRA1A, ADRB3, AKT1, ARPC1A, ARPC1B, ARPC3, BCL10, CCR1, CCR4, CELSR3, CRKL, CXCR6, FCER1G, FFAR4, GHSR, GPR156, GPR179, GPR61, GPR62, GPR65, GPR85, HCK, ITGA1, ITGA11, ITGA2, ITGA4, ITGAD, ITGAL, ITGAM, ITGAX, ITGB2, ITGB5, ITGB6, ITPR1, ITPR2, LPAR3, LPAR5, MAP2K5, MAP2K6, MRC2, MYL4, MYL6, MYLK, NTSR2, P2RY1, P2RY6, PIK3CD, PIK3CG, PIK3R2, PLAAT1, PLAAT3, PLB1, PLD5, PRDX6, PRKCB, PTGDR, PTGER3, PTGFR, PTK2, RAC2, RALB, RXFP1, S1PR4, SCARA3, SCARA5, SLC52A2, SMO, SPHK1, SYK, TIMD4*

### Presence of endothelial cells, pericytes, and smooth muscle cells in adipose tissues of dairy cows

We identified two subtypes of endothelial cells (EC), both expressing the EC signature gene *PECAM1* (Figure 6G)*.* EC1 subtype had a pronounced expression of classic endothelial cell markers, such as *VWF*, *CD300LG*, *ADAMTS9*, *TM4SF1, ACKR1,* and *TFPI* (Figure 6G)*.* Expression of *FABP4* and *CD36,* which are expressed in AT microvascular endothelial cells and involved in endothelial fatty acid handling machinery (Briot et al., 2018), was also abundant in EC1 (Supplementary Table S1). SAT had twice as many vascular EC1 than VAT (12.5% vs. 6.0%) (Figure 1G), and an increased expression of the FOS family genes (*FOSB* and *FOS*), *EGR1*, *ACKR,* a chemokine receptor, and *CD74*, a cell-surface receptor for the cytokine macrophage migration inhibitory factor, which may be observed in activated endothelial cells (Naeim, 2008). In contrast, VAT EC1 had increased expression of complement genes (*C3, C1QA,* and *CFB*) and bovine major histocompatibility complex (*BOLA*) (Supplementary Table S13). An activation of immune response and complement activation pathways was observed in VAT EC1 when compared to SAT EC1 (Supplementary Table S13).

In contrast to EC1, EC2 cells had increased expression of *MMRN1*, *LYVE1*, *CCL21*, and *PROX1*, markers of lymphatic endothelial cells. These results indicate the presence of lymphatic vasculature in AT of dairy cows, although considerably less pronounced when compared to vascular endothelial cells (0.3% vs. 10.1% of total nuclei). Lymphatic endothelial cells (EC2) expression profile was similar between depots (Supplementary Table S13).

Pericytes and smooth muscle cells (PE/SMC, Figure 2A) were identified based on the expression of pericytes (*NOTCH3* and *PDGFRB*) and smooth muscle cells marker genes (*ACTA2* and *MYL9*) (Figures 2B,C). Across depots, PE/SMC cells were not greatly abundant in dairy cows AT (2.75% of total nuclei; Figure 2D). When contrasting depots, we observed that SAT had a greater proportion of PE/SMC than VAT (3.2% vs. 2.0% of total nuclei; Figure 1G). An overall comparison on the gene expression between VAT and SAT PE/SMC (Supplementary Table S14) showed an increased expression of *C3, C1Qa, C1Qc, CFB*, and *LPL,* while a decreased expression of *FOS, FOSB*, and *EGR1* of VAT PE/SMC when compared to SAT PE/SMC.

## Discussion

In this study, we used both snRNA-seq and bulk RNA-seq to better understand the heterogeneity and depot-specific characteristics in AT of dairy cows. We have summarized the gene profile of each cell type and subtype in VAT and SAT of dairy cows revealed by our study in Graphical abstract. Part of the different cell types and depot-specificities characterized in the present study have been previously reported in mice and human models (e.g., Vijay et al., 2020; Emont et al., 2022; Strieder-Barboza et al., 2022). Compared with human AT single-cell databases, we observed that AT from dairy cows is as diverse as human SAT and VAT with similar cell types and subtypes, including numerous ASPCs, EC, and immune cells, and VAT-specific ME. These similarities open opportunities for using dairy cows as a model to study comparative human diseases that are associated with AT dysfunction. However, how the distinct cell subtypes contribute to the pathogenesis of diseases in dairy cows are yet to be studied.

Both snRNA-seq and bulk RNA-seq results showed consistent upregulation of HOX genes in SAT of dairy cows. HOX genes are known as a subset of the homeobox family transcription factors that play a key role during the differentiation of a variety of mammalian tissues. In humans, HOX genes regulate *in vivo* and *in vitro* adipogenesis (Cowherd et al., 1997; Cantile et al., 2003), and have depot-specificities (Yamamoto et al., 2010; Ahn et al., 2019). *PPARG* modulates different HOX genes in the AT, such as *HOXD4* (Kumar et al., 2021), which in our study was greatly expressed in SAT when compared to VAT (Supplementary Table S5). In ruminants, HOX and HOX-related genes play potential roles in regulating regional fat distribution in fat-tailed sheep (Kang et al., 2017). Different to our results, in which *HOXA9* was greatly expressed in abdominal SAT, in fat-tailed sheep, there was a downregulation of *HOXA9* expression in thoracic SAT when compared to perirenal VAT and tailhead SAT. Interestingly, while our bulk RNA-seq revealed that *HOXA10* and *HOXC10* expression was unique to SAT in dairy cows, in fat-tailed sheep, these two genes were expressed in all evaluated depots, but their relative expression level was greater in tailhead SAT than thoracic SAT and perirenal VAT (Kang et al., 2017). Overall, only scarce studies report potential roles of HOX genes, thus highlighting the need for further studies elucidating depot-specificities and role of HOX genes in AT function and metabolism.

One of the most evident depot-differences observed in our study was the greater expression of *C3* and other complement genes in VAT relative to SAT. Among the 13 clusters identified in our data, 11 had a greater expression of *C3* in VAT compared to SAT. *C3* was among the 5 most DEG (>Log_2_FC and *Adj p value* < 0.05) in most of these clusters. Studies reporting the roles of complement system in dairy cows’ AT are scarce (Zachut and Contreras, 2022) and C3 expression data are only available in subcutaneous AT samples (Zachut et al., 2018; Takiya et al., 2019; Salcedo-Tacuma et al., 2020). For example, Salcedo-Tacuma et al. (2020) reported upregulation of genes encoding the complement proteins C3 in postpartum dairy cows, while Zachut et al. (2018) showed an enrichment of complement pathway in AT of dairy cows that lost body weight intensively postpartum compared with cows that lost weight less intensively. These findings highlight a potential relationship between the expression of complement system genes and proteins with a pro-inflammatory, pro-lipolytic and pro-oxidative status in periparturient dairy cows. In humans, the increased expression of complement system genes, especially *C3*, has been correlated with metabolic dysfunction, including insulin resistance, inflammation, obesity, and diabetes (Engström et al., 2005; Barbu et al., 2015; Moreno-Navarrete and Fernández-Real, 2019). Gabrielsson et al. (2003) report a 4-fold increase in *C3* mRNA levels in omental VAT compared with abdominal SAT in obese male subjects, and suggest that C3 produced by the AT, especially omental VAT, as a key contributor to the plasma pool of C3, further corroborating the role of AT in systemic metabolism and inflammatory response.

During adipogenesis, adipose stem and progenitor cells (ASPC) proliferate (hyperplasia) and then accumulate lipids, increasing in size (hypertrophy). Both the increased number of adipocyte progenitors and the increase in intracellular lipids enhance AT adipogenic capacity. Hyperplastic growth is critical for a proper AT function and overall metabolic health (Ghaben and Scherer, 2019; Merrick et al., 2019). In dairy cows, a potential inability of AT to buffer excess fatty acids released into the bloodstream during the periparturient period could trigger other metabolic and inflammatory disease (Contreras et al., 2017; Contreras et al., 2018). Although adipogenesis is a central metabolic process in the AT, the definition of ASPC and its markers genes are not unanimous across literature even among research studying the same species. Moreover, the cellular hierarchy and biological mechanisms dictating ASPC differentiation are not yet completely understood (Merrick et al., 2019). The gene expression of commonly used ASPC markers by previous studies in human and mice models, e.g., *DPP4, CD142, CDF, TM4SF1,* and *CD34,* was negligible or absent in our dairy cow snRNA-seq database. In our study, we primarily identified ASPC subtypes based on the expression of *PPARG*, gene expressed during early ASPC differentiation, and *PDGFRA*, an ASPC marker used by different murine and human experiments (Merrick et al., 2019). In our study, we observed an increased abundance in overall ASPCs in SAT vs. VAT, as well as in the adipogenic-ASPC subtype, which greatly expressed genes that are upregulated during adipocyte differentiation, such as *PPARG* and *FABP4*. This profile is typical of ASPCs that are “committed preadipocytes” (e.g., Sun et al., 2020; Emont et al., 2022), meaning these cells are poised to differentiate into mature adipocytes. This contrasts with recent snRNA-seq data in human subjects with obesity (Strieder-Barboza et al., 2022), and may reflect the limited hyperplastic capacity of obese AT, which is more likely to expand through adipocyte hypertrophy (Ghaben and Scherer, 2019). Increased SAT adipogenic capacity in dairy cows might be fundamental in offsetting negative metabolic consequences of excessive concentrations of circulating free fatty acids during early postpartum of high producing dairy cows (Yung and Tak Mao, 2007; Gupta and Gupta, 2015). In contrast, defects in AT adipogenic capacity are associated to fibrosis and inflammation in human dysfunctional AT (Gyllenhammer et al., 2016). Our snRNA-seq data identified two subtypes of FAP ASPCs, which have the capability to differentiate into adipocytes or activated fibroblasts increasing ECM deposition (Contreras et al., 2021; Dohmen et al., 2022). Adipogenesis is inseparable from fibrogenesis due to closely related developmental origins of adipocytes and fibroblasts. Fibrogenesis refers to the generation of fibroblasts and their synthesis of proteins composing the ECM (Miao et al., 2016). In humans, fibrosis is defined as an excessive accumulation of ECM, such as collagens, which can result from an imbalance between excess synthesis ECM components and an impairment in degradation of these proteins. Thus, increased fibrogenesis can contribute to the development of fibrosis. Our data revealed a pro-fibrogenic/fibrotic potential of ASC3, with upregulation of fibrosis genes (collagens, *FN1, DCN, FBLN1, MMP2, LUM, SPARC,* and *LOX*) (Divoux et al., 2010). Additionally, ASPC3 demonstrated a pro-inflammatory potential with upregulation of *CCL2, CXCL3, C3, C1S,* and *PTGS2.* This profile overlaps with human VAT ASPCs which are positively correlated with insulin resistance (Vijay et al., 2020) and with mice AT fibro-inflammatory progenitors (FIPs), which also had an anti-adipogenic function (Hepler et al., 2018). Additionally, recent work reported a FAP ASPC subtype in obese human AT with greater adipogenic capacity and lipolytic responses compared with an inflammatory mesothelial-like ASPC subtype (Strieder-Barboza et al., 2022). These findings agree with previous studies identifying increased FAP ASPCs in obesity and reported a close association of FAPs with adipose tissue fibrosis (Marcelin et al., 2017) and type 2 diabetes (Vijay et al., 2020) in human obesity. Overall, these findings suggest that, as in humans and mice (Hepler et al., 2018; Min et al., 2019), bovine AT contains transcriptionally diverse ASPCs which may regulate adipogenesis, inflammation, and fibrosis in a depot-specific manner.

Recently, numerous studies using different species aimed at determining the source and function of mesothelial cells (ME) in the AT, with contrasting results. Mesothelial cells derive from mesoderm, express mesenchymal features, and form a monolayer over visceral and parietal surfaces of the peritoneal, pleural, and pericardial cavities (Yung and Tak Mao, 2007; Gupta and Gupta, 2015). Unanimously, literature has demonstrated a striking depot-specific pattern in AT ME, which are VAT-specific (e.g., Chau et al., 2011; Vijay et al., 2020; Emont et al., 2022). In our study, SAT preadipocytes and adipocytes expressed mesothelial markers *in vitro*, but only 0.3% of SAT nuclei were annotated as ME, thus highlighting a strong depot-specificity of ME in VAT of dairy cows. Current literature is contradictory about whether mesothelial cells are true adipocyte progenitors. In humans, VAT-derived ME-like progenitors exhibited pronounced expression of omentin (*ITLN1*) and mesothelin (*MSLN*), while SAT progenitors expressed *CFD* (Vijay et al., 2020). Chau et al. (2014) observed that *Wt1* positive cells can differentiate into adipocytes, muscle cells, and osteoblasts in mice. While *Wt1* ^*+*^ ME are considered VAT-specific ASPCs that become adipocytes (Chau et al., 2014), *Krt19* ^*+*^ ME did not have adipogenic capacity (Westcott et al., 2021). In our study, all ME expressed *MSLN* and *KRT19*. Even though we did not test the adipogenic capacity of ME, we observed an increased expression of *CD34* and *IGF2*, which are known markers of ASPCs and adipocytes in *WT1* ^*+*^ *UPK3B* ^*+*^ ME2. In fact, our flow cytometry data revealed that a proportion of MSLN^+^ cells correspond to the ASPC population (CD31^−^CD45^−^), while others co-express MSLN and the endothelial cell marker, CD31 (Figure 4G). Consistently, Westcott et al. (2021) report that *Wt1* expression in mice AT is not exclusive to visceral adipose mesothelium, but also expressed in a population of *Pdgfra +* preadipocytes, which can originate adipocytes. Our snRNAseq analysis revealed an exclusive expression of *WT1* in ME, although around 15% of these cells also expressed *PDGFRA*. A previous study (Westcott et al., 2021) reports that only *WT1* ^*+*^ *PDGFRA* ^*+*^ cells present in the ME cluster can differentiate into adipocytes, while the *WT1* ^*+*^ *PDGFRA* ^*-*^ fraction cannot. Recent data also describe a pro-inflammatory and low-adipogenic potential of VAT-specific mesothelial cells in human obesity (Strieder-Barboza et al., 2022). To better understand whether ME are adipocyte progenitor cells in dairy cows, ME cells should be isolated from AT and their adipogenic capacity evaluated in different *in vitro,* conditions, such as the use of basal medium with and without and adipogenic inducers (i.e., insulin, PPARG agonists, or thiazolidinediones).

In comparison to scRNA-seq, snRNA-seq allows the sequencing of different cell types regardless of their size. When considering AT analysis, this advantage is substantial due to the large size of mature adipocytes. For that reason, studies revealing mature adipocyte diversity at a single-nuclei level are scarce and have never been previously reported for dairy cows. Adipocytes were generally considered to be monotypic and homogeneous in function (Emont et al., 2022); however, recent evidence shows otherwise (Min et al., 2019; Sárvári et al., 2021; Emont et al., 2022), including the present study. In our study, adipocyte (AD) was the most abundant cell type in both SAT and VAT. We identified four transcriptionally distinct AD subpopulations in both depots, and the analysis of their gene expression suggest similarities with different ASPC subpopulations, consistent with recent studies using snRNA-seq that identified diverse adipocyte subtypes with marked depot-specificities in human AT (Emont et al., 2022; Strieder-Barboza et al., 2022). These findings might translate into distinctive adipocytes origins and metabolic functions in AT of dairy cows. Furthermore, an overall comparison between mature adipocytes in SAT vs. VAT revealed an increased expression of adipogenic and lipogenic genes in SAT and an increased expression of lipolytic genes in VAT. In humans, VAT has been characterized for having greater lipolytic rate than SAT (Arner, 1995; Smith and Zachwieja, 1999), although SAT lipolysis contributes substantially to circulating lipid levels since it’s the body’s largest fat depot (Rydén and Arner, 2017). In dairy cows, few studies have compared SAT and VAT metabolism in different energy balance and lactation stages, possibly due to the difficulty to access VAT in comparison with SAT. However, the available literature corroborates with our findings. For example, in cows with intensive lipolysis, fatty acid profile of plasma non-esterified fatty acids (NEFA) showed remark similarity with fatty acid profile of VAT (Hostens et al., 2012), and AT mass mobilization showed to be greater in VAT than SAT (Ruda et al., 2019).

Vascular endothelial cells, mononuclear phagocytes, and lymphocytes were among the different cell populations in which complement system genes (e.g., *C3* and *CFB*) were greatly expressed in VAT than SAT. Different stimuli has been reported to induce the production of complement protein by these cell types. For example in humans, synthesis of C3 by macrophages is increased upon stimulation with acetylated low-density lipoprotein, oxidized low-density lipoprotein, IgA, or IgG immune complexes (Laufer et al., 1995; Mogilenko et al., 2012), while INF-gamma induced synthesis of different proteins of complement system in human endothelial cells (Ripoche et al., 1988). In our study, although the percentage of immune and vascular endothelial cells expressing pro-inflammatory cytokines, such as IL6 and IL1a, were considerable low (<1%), other results indicate a greater inflammatory status of VAT when compared to SAT, which could help us explain the high contrast in the expression of complement system genes between depots.

We observed greater proportion of MAC in VAT than SAT. These results corroborate with studies in dairy cows, humans, and mice that revealed greater macrophage infiltration in visceral depots (Weisberg et al., 2003; Harman-Boehm et al., 2007; Akter et al., 2012). Contreras et al. (2015) observed that macrophage infiltration in the AT of dairy cows is associated with metabolic disease (hyperketonemia, increased concentration of blood NEFA, and displaced abomasum) in early postpartum dairy cows; authors reported a significantly higher number of SVF cells expressing macrophage-specific cell surface markers in omental compared with subcutaneous AT (Contreras et al., 2015). We observed leukocyte activation and migration, immune and inflammatory response, phagocytosis, among others biological pathways in VAT immune cells when compared to SAT. Bulk-RNAseq analysis confirmed the upregulation of immune response and pro-inflammatory regulators in VAT vs. SAT and the activation of pathways associated with increased inflammation underlined by T-lymphocytes, NK cells, and macrophages (Table 1), consistent with our snRNAseq results. Our findings are also in line with previous studies in different species reporting enhanced inflammation in VAT. In non-pregnant non-lactating Holstein dairy cows, AT transcript profiles showed that in comparison with SAT, mesenteric VAT had an increased pro-inflammatory response (Moisá et al., 2017). Similarly, in human subjects, VAT has greater pro-inflammatory characteristics, presenting the double proportion of pro-inflammatory macrophages when compared to SAT (Kralova Lesna et al., 2016).

Further analysis of MAC revealed five individual subpopulations with different expression profiles and possible different functions in the AT. For example, we identified a MAC subpopulation (MAC4) enriched with lipid metabolism genes such as *LIPA, LPL, CD36*, *FABP4, CD9, LGALS1,* and *LGALS3* (e.g., Jaitin et al., 2019; Vijay et al., 2020). Jaitin et al. (2019) observed that a population of macrophages expressing lipid-associated genes arises during obesity and are named lipid-associated macrophages (LAMs). Interestingly, LAMs have important function in metabolic homeostasis in a *TREM2-*dependent manner, buffering the excess lipids accumulated during the development of obesity (Chen et al., 2021). In contrast, loss of *TREM2* seems to prevent LAM formation causing adipocyte hypertrophy, weight gain, and insulin resistance (Jaitin et al., 2019; Worthmann and Heeren, 2020). In the present study, however, only around 8% of MAC4 cells expressed *TREM2*. Based on these previous reports, we speculate that 1) the majority of MAC4 cells in our study do not present the effects on metabolic homeostasis as the ones characterized in human and mice, or 2) factors other than *TREM2* may affect the function of lipid-associated macrophages in dairy cows, that greatly expressed the genes necessary to excessive lipids handling regardless of *TREM2* expression level. Higher presence of MAC4 in VAT of dairy cows might also indicate a greater necessity of fatty acid buffering in the visceral AT. Overall, we emphasize that further studies are necessary to elucidate the specifics of these immune cell subpopulations, especially in determining their pro or anti-inflammatory phenotypes and their role in AT dysfunction.

In addition to MAC, the most prevalent immune cells observed in our study, we also characterized a less abundant immune population of NKT cells. In agreement with our experiment, other studies using snRNA-seq have reported the presence of natural killer and T cells in the AT of humans and mice in lower proportions when compared to MAC (Emont et al., 2022; Strieder-Barboza et al., 2022) Although these natural killer and T cells represented the rarest cell type in the AT of dairy cows and their characterization in subtypes was not feasible in our database, their presence in the tissue is particular of notice. NK and T cells are lymphoid cells, and recently have been gaining notoriety due to their important regulatory role in the AT. Through the production of cytokines and influencing macrophages polarization, distinct populations of these immune cells can either improve metabolic homeostasis or contribute with metabolic disorders (Vivier et al., 2008; Lee et al., 2016; Wang and Wu, 2018; Ferno et al., 2020).

Endothelial cells represented around 10% of all cells in the AT of dairy cows. Further analysis revealed distinct gene expression and the presence of two different populations: vascular and lymphatic EC. Vascular EC was predominant in abundance compared to lymphatic EC. Interestingly, vascular EC expressed genes involved in lipid metabolism (e.g., *FABP4* and *CD36*), which might corroborate with studies suggesting vascular endothelium can originate mature adipocytes (Tran et al., 2012; Min et al., 2016). However, further studies are necessary to demonstrate this hypothesis in dairy cows’ AT. Studies also suggest that ECs play essential role in the maintenance of fatty acid fluxes and inflammatory response in the AT (Briot et al., 2018). Lymphatic ECs have been recently shown as an important link between lymphatic vessels and AT, with a bilateral relationship between lymphatic dysfunction and occurrence of obesity and fat accumulation (Escobedo and Oliver, 2017). In our study, lymphatic EC represented only 0.3% of total nuclei and differences between depots were not observed. However, it is interesting to highlight that lymphatic EC were present in both SAT and VAT, in contrast to a study by Vijay et al. (2020), in which authors revealed a strong visceral depot-specificity.

Limitations of our study include limited batch size, unknown health status of sampled animals, and the relative low number of total nuclei sequenced compared with previous studies in mice and human models. Moreover, absence of similar studies in bovine or other ruminant species makes it particularly challenging to correlate some of our findings.

Adipose tissue analysis at a single-cell and single-nuclei level allows targeted gene expression changes within specific cell populations, lineage dynamics, and mechanisms governing the development and function of adipocytes in a depot-dependent manner. In summary, for the first time, we demonstrated depot-specific heterogeneity at a single-nuclei level in VAT and SAT of dairy cows. Our data suggest that revealing transcriptionally and functionally distinct depot-specific cell types is a promising step towards elucidating mechanisms linking AT dysfunction and the occurrence of metabolic diseases in dairy cows, which could then guide us to define targeted approaches to prevent their occurrence at a farm level.

## Data Availability

The datasets presented in this study can be found in online repositories. The names of the repository/repositories and accession number(s) can be found below: https://www.ncbi.nlm.nih.gov/geo/, GSE211707.
