# Supplementary material for: Single-nuclei analysis reveals depot-specific transcriptional heterogeneity and depot-specific cell types in adipose tissue of dairy cows
Source: Front Cell Dev Biol. 2022 Oct 14;10:1025240. doi: 10.3389/fcell.2022.1025240 (PMC9616121; doi:10.3389/fcell.2022.1025240)
Supplement: Supplementary file 2 [file Table1.docx]

Supplementary Material

# Supplementary Figures and Tables

## Supplementary Figures

**Supplementary Figure 1.** **Outline of experimental workflow**. **(A)** Visceral adipose tissue (VAT) was collected from greater omentum and subcutaneous adipose tissue (SAT) from the right flank of the same three lactating Holstein dairy cows. **(B)** VAT and SAT samples were dissociated in a dounce homogenizer and **(C)** nuclei pellet isolated, stained with trypan blue solution (0.4%) and visualized by phase-contrast light microscopy. **(D)** Single nuclei were purified using flow cytometry with fluorescence activated cell sorting (FACS). Sorting strategy included doublet discrimination and selection of intact nuclei by sub-gating on PI+ stanning. **(E)** GEMs (Gel Bead-In Emulsions) and library construction according to 10X Genomics guidelines. **(F)** Pooled libraries were subjected to 150 bp paired-end sequencing according to the manufacturer’s protocol (Illumina NovaSeq 6000). **(G)** Data analysis was performed using the scRNA-seq package Seurat v3.1.4 in the R environment (version 4.1.3).

## Supplementary Tables

**Supplementary Table 1.** Top genes expressed in clusters of dairy cows’ adipose tissue-derived cells from 6 samples (3 SAT and 3 VAT).

**Supplementary Table 2.** GO analysis according with different cell types or overall comparison between VAT and SAT.

**Supplementary Table 3.** GO analysis of different adipose stem and progenitor cells (ASPC) populations.

**Supplementary Table 4.** GO analysis of different mature adipocytes (AD) populations.

**Supplementary Table 5:** Different expressed genes between VAT and SAT.

**Supplementary Table 6:** Top genes expressed in different subpopulations of adipose stem and progenitor cells (ASPC).

**Supplementary Table 7:** Different expressed genes between VAT and SAT adipose stem and progenitor cells (ASPC).

**Supplementary Table 8:** Top genes expressed in different subpopulations of mature adipocytes (AD).

**Supplementary Table 9:** Different expressed genes between VAT and SAT mature adipocytes (AD).

**Supplementary Table 10:** Top genes expressed in MAC subclusters (MAC1-5).

**Supplementary Table 11:** Different expressed genes between VAT and SAT immune cells.

**Supplementary Table 12:** Different expressed genes between overall VAT and SAT macrophages and monocytes (MAC) and subpopulations.

**Supplementary Table 13:** Different expressed genes between overall VAT and SAT endothelial cells (EC) and different populations.

**Supplementary Table 14:** Different expressed genes between overall VAT and SAT pericytes and smooth muscle cells.

**Supplementary Table 15:** GeTMM values from bulk-RNA sequencing analysis of VAT (n=3) and SAT (n=3) samples.

**Supplementary Table 16:** Differentially expressed genes (robustFDR<0.05) based GeTMM values from bulk-RNA sequencing analysis of VAT (n=3) and SAT (n=3) samples.
