# Supplementary figures and images for: Single-nuclei analysis reveals depot-specific transcriptional heterogeneity and depot-specific cell types in adipose tissue of dairy cows
Source: Front Cell Dev Biol. 2022 Oct 14;10:1025240. doi: 10.3389/fcell.2022.1025240 (PMC9616121; doi:10.3389/fcell.2022.1025240)

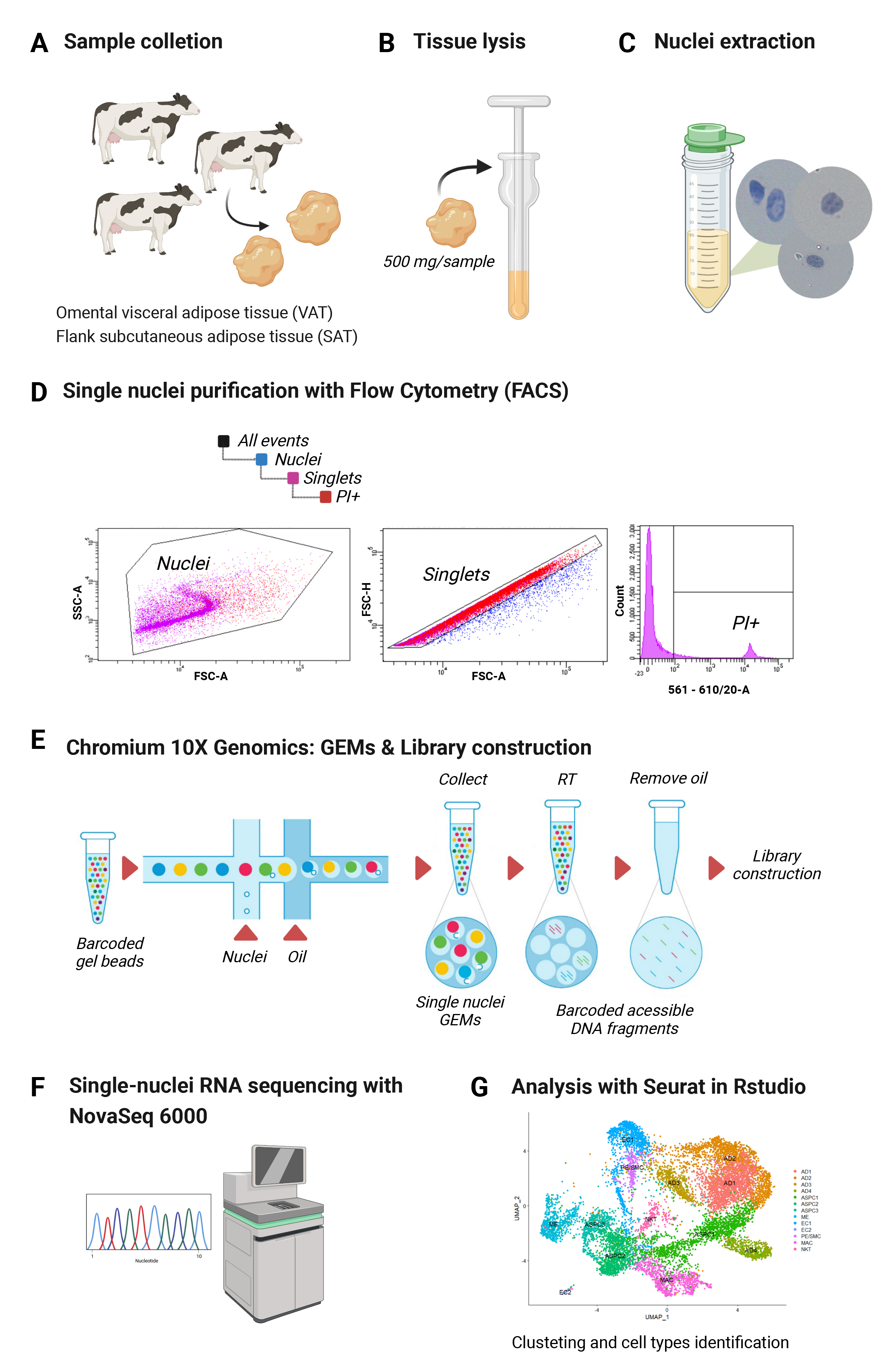

Supplement: Supplementary file 3 [file Image1.TIF]
